# Presence of host and bacterial-derived collagenolytic proteases in carious dentin: a systematic review of ex vivo studies

**DOI:** 10.3389/fcimb.2023.1278754

**Published:** 2023-10-31

**Authors:** Cecília de Brito Barbosa, Isabela Monici Silva, Jéssica Alves de Cena, Cristine Miron Stefani, Naile Dame-Teixeira

**Affiliations:** Department of Dentistry, School of Health Sciences, University of Brasília, Brasília, Brazil

**Keywords:** dentin caries, root caries, collagenase, microbial collagenase, host collagenase, systematic review

## Abstract

**Introduction and aim:**

The presence of host collagenases in the degradation of the protein matrix at later stages of carious dentin lesions development, as well as the potential involvement of bacterial collagenases, have been suggested but lack conclusive evidence. This study aims to conduct a systematic review to comprehensively assess the profile of host and bacterial-derived collagenolytic proteases in both root and coronal dentin carious lesions.

**Methods:**

The search was performed in eight databases and the grey literature. Studies evaluating *ex vivo* dentin, extracted teeth, or biofilms from natural caries lesions were included. The methodological quality of studies was assessed using the Joanna Briggs Institute tool. Synthesis of the results and the certainty of evidence were performed following the Synthesis without Meta-analysis (SWiM) checklist and GRADE approach for narrative synthesis, respectively.

**Results:**

From 935 recovered articles, 18 were included. Although the evidence was very uncertain, it was possible to suggest that 1) MMP-2, MMP-9, MMP-13, and CT-B may be increased in carious dentin when compared to sound dentin; 2) there is no difference in MMP-2 presence, while MMP-13 may be increased in root when compared to coronal carious dentin; 3) there is no difference of MMP-2 and MMP-9 expression/activity before and after cavity sealing; 4) MMP-8 may be increased in the dentin before cavity sealing compared to dentin after cavity sealing; 5) there is no difference of MMP-20 in irradiated vs. non-irradiated carious dentin. MMP-20 probably reduces in carious outer dentin when compared to carious inner dentin (moderate certainty). Genes encoding bacterial collagenolytic proteases and protein-degrading bacteria were detected in coronal and root carious lesions.

**Conclusion:**

Trends in the direction of the effect were observed for some collagenolytic proteases in carious dentin, which may represent a potential target for the development of new treatments. (Protocol register-PROSPERO: CRD42020213141).

## 1 Introduction

Untreated carious lesions at the cavity level are a widespread issue affecting a significant portion of the global population (Kassebaum et al., 2015), which has substantial impact on individual’s quality of life (Severo Alves et al., 2013). Moreover, there is a growing incidence of root caries (Kassab and Cohen, 2003; Griffin et al., 2004). Both cavitated and root caries lesions share several key features, particularly the involvement of dentin. To develop effective strategies for prevention and control, it is crucial to explore the etiology of the carious process in both coronal and root dentin caries, especially given the limited attention dentin lesions have received compared to enamel lesions.

The issue has gained significance with the emergence of the ecological hypothesis of dentin and root caries (Takahashi and Nyvad, 2016), which suggests that the demineralization in the dentin caries is followed by a proteolytic stage, leading to the degradation of the exposed organic collagenolytic matrix (Takahashi and Nyvad, 2016). This degradation of dentin’s collagen matrix occurs after exposure to collagen fibrils due to the low pH triggered by the bacterial acidogenesis (Tjäderhane et al., 1998; Tjäderhane et al., 2015). During demineralization, there is also some breakdown of the dentin matrix cross-links within the dentin matrix (Dayan et al., 1983). Consequently, the type I collagen became susceptible to proteolysis by collagenolytic enzymes, which, when activated, can site-specifically cleave its molecules (Tjäderhane et al., 2015). Understanding these molecular processes is crucial for expanding targets for new interventions.

Building this understanding, it has been proposed that host collagenases play a role in the proteolytic stage of carious dentin lesions (Tjäderhane et al., 1998; Nascimento et al., 2011; Vidal et al., 2014). Some studies have shown that matrix metalloproteinases are present within the dentinal organic matrix, which may become activated under acidic conditions, typically around pH 4.5. Subsequently, during the remineralization phase of the pH cycle, when the pH is neutralized, collagen degradation could occur (Kawasaki and Featherstone, 1997; Chaussain-Miller et al., 2006; Tjäderhane et al., 2015; Takahashi and Nyvad, 2016). However, it’s worth noting that most of these studies have also detected these proteases in the sound dentin matrix. Despite the debate on the presence of endogenous and salivary proteases in dentin organic matrix degradation in critical reviews (Chaussain-Miller et al., 2006; Tjäderhane et al., 2015), robust evidence regarding their presence in dentin and root caries remains elusive. Much of the existing evidence relies on *in vitro* models, which may only partially mimic the natural development caries lesions (Tjäderhane et al., 2015).

When the bacterial collagenases are under debate, the situation is far less clear. There is a relatively limited body of literature addressing a microbial role in this process, mostly *in vitro* or *in situ* study designs. Studies have indicated that bacterial collagenases may not withstand the acidic pH drop (pH 4.3) during the demineralization phase of a pH cycling model (Kawasaki and Featherstone, 1997). This observation supports the hypothesis that they do not play an important role in the carious process. Such approaches, however, have failed to address bacterial collagenolytic proteases in their natural system could be activated in the remineralization phase during the pH cycles, similarly to host collagenases. Furthermore, complex biofilms and interconnected metabolisms between different members of dental biofilms may activate collagenases in ways that are not readily detectable *in vitro*. It is important to note that early reports suggesting that the predominant microorganisms are not capable to degrade collagen matrix in cavitated caries lesions may be outdated, as they were conducted prior to the application of Next-Generation Sequencing (NGS) technologies in the field of oral microbiology, primarily testing culturable bacteria (Kawasaki and Featherstone, 1997; Tjäderhane et al., 1998).

To gain a more comprehensive understanding of the factors influencing the activation of various proteases, including variations in pH, the role of complex microbiota, and potential substrates, it would be valuable to conduct studies evaluating *ex vivo* natural caries lesions. Given the advancements in our knowledge of this area over the past few decades, a systematic review could consolidate the current evidence and pave the way for innovative caries preventive strategies centered on collagenases as potential targets for new drugs (Chaussain-Miller et al., 2006). With this context in mind, the aim of this study was to conduct a systematic review to comprehensively assess the profile of host and bacterial-derived collagenolytic proteases in both root and coronal dentin carious lesions. This knowledge synthesis may contribute to a deeper understanding of the role of these proteases in the development and progression of caries, potentially opening up new avenues for preventive strategies and treatment approaches.

## 2 Materials and methods

### 2.1 Eligibility criteria

The acronym PECO (Population; Exposure; Comparator; Outcomes) was used to design the research question:

Participants/population: human dental tissue or biofilms collected from human dental tissue (*ex vivo* samples*);*
Exposure: caries in dentin and/or root surface;
Comparator/control: sound dentin/no control/sealed dentin;
Outcome: collagenolytic (or gelatinolytic/collagenolytic protease) presence/activity/gene expression.

#### 2.1.1 Inclusion criteria

Studies eligible for this review were the ones analyzing human dentin or biofilms with natural caries lesions. These comprised *ex vivo* extracted teeth or clinical carious/biofilm samples from observational (cross-sectional, case-control, or cohort studies), and experimental studies (randomized or non-randomized clinical trials), with no limitation of publication year.

#### 2.1.2 Exclusion criteria

Exclusion criteria were the following: (1) reviews, letters, conference abstracts, personal opinions, book chapters, and protocols; (2) *in vitro* studies, including artificial caries models; (3) studies performed in dental enamel caries/erosion lesion; (4) studies evaluating only sound dentin; (5) studies evaluating other than enzymatic activity of collagenases/gelatinases/collagenolytic proteases; (6) studies written in the non-Latin alphabet; and (7) animal studies. Although not foreseen in protocol phase, studies on matrix metalloproteinases (MMPs) inhibitors were not included after the full-text reading due to the particularities of their research questions.

### 2.2 Data sources and search strategy

The search was performed in March 2022. **Supplementary Appendix 1** shows the complete search strategy. “Dental caries” and “Collagenases”, their synonyms and variations were used as main search terms for PubMed search strategy elaboration, which was adapted for each electronic database: MEDLINE via PubMed, LILACS, Web of Science, Scopus, Cochrane, EMBASE, and Livivo. Gray literature search was also performed in Google Scholar, ProQuest (Dissertations and Thesis), and OpenGrey. Reference lists from included studies were assessed to identify other potentially eligible studies. No language or interval time restrictions were applied. Duplicates were identified through EndNoteWeb (Clarivate Analytics, Mumbai) and then manually identified on Rayyan QCRI^®^ (Qatar Computer Research Institute, Qatar).

### 2.3 Study selection and data extraction

Two independent reviewers (IMS and CBB) selected the included articles. First, both reviewers independently read titles and abstracts, applying the eligibility criteria within a web application tool designed for systematic reviews (Rayyan QCRI^®^, Qatar Computing Research Institute). In a second stage, the same reviewers performed a full-text reading while applying for the eligibility criteria. In both stages, all the retrieved information was crosschecked by a third reviewer (JAC). Once a study was selected for the second stage and it was not available in any way through online sources, the corresponding author was contacted to provide the full-text.

### 2.4 Methodological quality assessment

The methodological quality assessment of the included studies was evaluated by two independent reviewers (IMS and CBB) using the JBI Critical Appraisal Checklist for Analytical Cross-Sectional Studies (https://jbi.global/critical-appraisal-tools). Despite all questions of the adopted appraisal tool are considered important, four of them were considered critical items to this systematic review due to the design of included articles. These items included: “Were the criteria for inclusion in the sample clearly defined?”; “Were the study subjects and the setting described in detail?”; “Was the exposure measured in a valid and reliable way?” and “Were objective, standard criteria used for measurement of the condition?”.

Criteria adopted for considering a low methodologic quality were: two or more “no” answers in those critical items; or one “no” and two or more “unclear” answers in those critical items; or one “no” answer in a critical and two or more “no” answers in non-critical items. High methodological quality was considered when a study gets a maximum one “no” answer or two “unclear” answers in non-critical items. Studies were considered with a moderate methodological quality when they did not fit the criteria, as described before (Dame-Teixeira et al., 2021).

### 2.5 Data analysis

Data analysis was performed according to the synthesis without meta-analysis (SWiM) reporting guideline (Campbell et al., 2020). Due to the high heterogeneity of the included studies, turning out a meta-analysis was unfeasible. The studies and their results were grouped and analyzed according to the origin of collagenase (host or bacterial-derived), the method used to detect collagenases presence or activity (collagenases assays, including western blots and commercial kits, gene expression, immunohistochemistry), and paired comparisons (sound vs. carious dentin; root vs. coronal dentin; outer vs. inner dentin; irradiated vs. non-irradiated dentin). The metrics to measure the outcome were the statistically significant differences related to the primary studies in collagenase presence/activity at the paired comparisons (effect direction).

The certainty of the evidence was addressed through the GRADE approach for narrative synthesis (in the absence of a single effect estimate) (Murad et al., 2017), considering the main five domains for downgrading (risk of bias, inconsistency, indirectness, imprecision, and likelihood of publication bias) for the main collagenases found for each paired comparison. When average differences between groups and standard deviations were available in the literature, the optimal information size (OIS) was calculated to determine the imprecision. Results were then standardly reported according to GRADE guidelines, based on the size of the effect and the certainty of the evidence (Santesso et al., 2020).

## 3 Results

**Figure 1:**
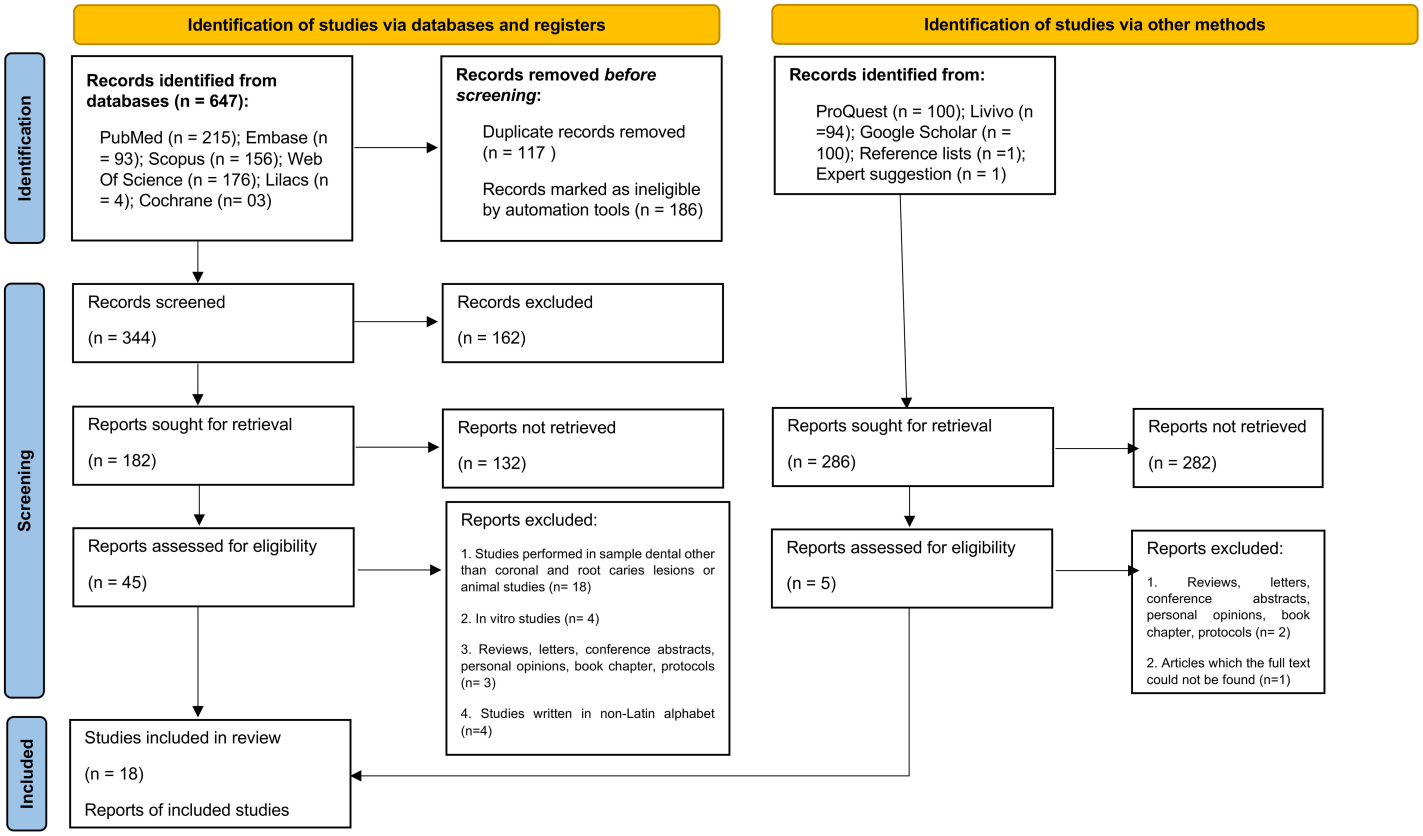
Flowchart of the study according to the PRISMA 2020 for systematic reviews which included searches of databases, registers and other sources.

A total of 935 records were found by searching the databases. After removing duplicates, 568 remained for titles and abstracts reading. **Supplementary Appendix 2** shows the excluded studies and reasons for exclusion. From 50 records selected for full-text reading, 18 studies were included for qualitative synthesis as they met the eligibility criteria (**Figure 1**; **Table 1**). Only four studies evaluated bacterial collagenases in dental caries samples, and all others were devoted to the study of host collagenases. While studies on bacterial collagenases evaluated mainly *ex vivo* biofilms using microbial nucleic acids analysis, studies on host collagenases mostly used immunohistochemistry direct to the dentin tissue, so that the host and bacterial-derived collagenolytic proteases could be differentiated. No studies reported both, host and bacterial collagenases.

**Table 1:** Characteristics and methodological quality of individual studies evaluating host collagenases according to the comparative analysis (N= 14; some studies are duplicated as presented results for more than one comparative analysis).

Author, year, country	Sample location and size	Type of collagenase	Method used to identify/quantify collagenases	Main results	Methodological quality
Sound vs. Carious dentin					
(Boushell et al., 2011); USA	Coronal; Caries (n=6) vs. sound (n=10)	MMP-2 and BSP	Immunohistochemistry	No differences between carious vs. sound or caries severity.	**+**
(Charadram et al., 2012); Australia	Coronal; Caries (n=30) vs. sound (n=15)	MMP- 2	Immunohistochemistry Collagenase assay	MMP-2 activity in the reactionary dentin was significantly higher than in the sound dentin.	**+++**
(Loreto et al., 2014); Brazil	Coronal; Caries (n=10) vs. sound (n=3)	MMP-13	Immunohistochemistry	**MMP-13 increased in caries**; very weak in sound dentin.	**++**
(Nascimento et al., 2011); Brazil	Coronal; Caries (n=8) vs. sound (n=4)	CT-B MMPs and Cysteine proteinases	Immunohistochemistry Collagenase assay	CT-B less intense in sound than carious dentin; MMPs and cysteine proteinase activity increase with the age; Cysteine proteinase increase with the lesion depth, higher in active than inactive lesions.	**++**
(Shimada et al., 2009); Japan	Coronal (n=5)	MMP-2, MMP-8, MMP-9 and MMP-20	Immunohistochemistry	MMP-2 present in all conditions, either sound or caries; **MMP-8 and MMP-9 increased at the outer carious dentin when compared to sound.**	**++**
(Toledano et al., 2010); Spain	Coronal and root (n=10)	MMP-2	Immunohistochemistry	MMP-2 present in all conditions, either sound or caries, increased closer to the wider dentinal tubules; Demineralized (“affected”) dentin exhibited a low intensity of MMP-2 (but higher than sound).	**+**
(Tjäderhane et al., 1998); Canada; Finland; England	Coronal (n = 37)	MMP-2, MMP-8 and MMP-9	Western blot enzymography	MMP-2, MMP-8 and MMP-9 were identified in carious dentin lesions; Overall, MMP-9 appeared to be the predominant gelatinolytic enzyme in dentin caries lesions.	**++**
(Vidal et al., 2014); Brazil	Coronal; Caries (n=5) vs. sound (n=5)	CT-B, CT-K and MMP-2	Immunohistochemistry	**CT-B, CT-K, MMP-2, and MMP-9 significantly higher in in caries than sound dentin.**	**+**
Root vs. Coronal					
(Yeon Lee et al., 2013); USA	Coronal and root; (n=7)	MMP-13	Collagenase assay	**MMP-13 increased in root caries**, and absent in coronal caries.	**++**
(Toledano et al., 2010); Spain	Coronal and root (n=10)	MMP-2	Immunohistochemistry	No differences root and coronal dentin.	**+**
Lesion depth and location					
(Ballal et al., 2017); Switzerland	Coronal (n=33)	MMP-2 and MMP-9	ELISA	**Significantly more MMP-9 in deep carious lesions than shallow;** No difference for MMP-2	**++**
(Boushell et al., 2011); USA	Coronal; Caries (n=6) vs. sound (n=10)	MMP-2 and BSP	Immunohistochemistry	MMP-2 and BSP in carious and sound dentin (**higher activity closer to odontoblastic processes**). Significantly more immunostaining for MMP-2 and BSP in caries-affected tubules.	**+**
(Shimada et al., 2009); Japan	Coronal (n=5)	MMP-2, MMP-8, MMP-9 and MMP-20	Immunohistochemistry	MMP-2 present in all conditions; **MMP-8 and MMP-9 increased at the outer carious dentin when compared to inner. MMP-20 reduced toward the outer caries.**	**++**
(Gomes-Silva et al., 2017a); Brazil	Coronal; Irradiated (n=19) vs. non-irradiated (n=17) carious dentin	MMP-2 and MMP-9	Immunohistochemistry	**MMP-9 predominantly positive in the non-irradiated**; MMP-2 and MMP-9 more expressed along the dentin-enamel junction, and highly positive in carious dentin.	**++**
(Gomes-Silva et al., 2017b); Brazil	Coronal; Irradiated (n=19) vs. non-irradiated (n=17) carious dentin	MMP-20	Collagenase assay	No differences, but more expressed along the dentin-enamel junction.	**++**
(Vidal et al., 2014); Brazil	Coronal; Caries (n=5) vs. sound (n=5)	CT-B, CT-K and MMP-2	Immunohistochemistry	MMP-2 is more expressed in carious dentin	**+**
Before vs. after cavity sealing					
(Chibinski et al., 2014); Brazil	Coronal (n=25)	MMP-2, MMP-8 and MMP-9	Immunohistochemistry	**Significant increase of MMP-8 before cavity sealing**	**++**
(Kuhn et al., 2016); Brazil	Coronal; (n=23)	MMP-2, MMP-8 and MMP-9	Immunohistochemistry	**Reduction of MMP-8 after 60 days of cavity sealing**.	**++**
Irradiated vs. Non - irradiated					
(Gomes-Silva et al., 2017b); Brazil	Coronal; Irradiated (n=19) vs. non-irradiated (n=17) carious dentin	MMP-20	Collagenase assay	**No significant difference between irradiated and non-irradiated.**	**++**

MMP, Metaloproteinase.

CT-B, Cathepsin B.

CT-K, Cathepsin K.+ = low methodological quality; ++ moderate methodological quality; +++ high methodological quality.

Due to the characteristic of the studies, low sample sizes were observed, ranging from 3 to 42 samples (on average 15 samples). As for the methodological quality, 4 studies were classified as “high” methodological quality (Simon-Soro et al., 2013; Bello Arroyo, 2016; Damé-Teixeira et al., 2018), 11 as “moderate”(Tjäderhane et al., 1998; Shimada et al., 2009; Hashimoto et al., 2011; Nascimento et al., 2011; Yeon Lee et al., 2013; Chibinski et al., 2014; Loreto et al., 2014; Kuhn et al., 2016; Ballal et al., 2017; Gomes-Silva et al., 2017a; Gomes-Silva et al., 2017b), and 3 as “low” methodological quality (Toledano et al., 2010; Boushell et al., 2011; Vidal et al., 2014) according to the JBI tool (**Tables 2**, **3**; **Supplementary Appendix 3**).

**Table 2:** Summary of the findings of the qualitative, quantitative, and certainty of the evidence produced analyses for MMPs in the included studies.

MMP	Studies included/Methodological quality	Caries Group (n)	Sound Group (n)	Studies/Findings	Certainty of evidence GRADE (see Appendix 6 for more details, Supplementary File 2)
MMP-2	(Tjäderhane et al., 1998) **++** (Vidal et al., 2014) **+** (Chibinski et al., 2014) **++** (Ballal et al., 2017)**++** (Gomes-Silva et al., 2017a) **++** (Shimada et al., 2009) **++** (Kuhn et al., 2016)**++** (Boushell et al., 2011) **+** (Toledano et al., 2010) **+** (Charadram et al., 2012; Shimada et al., 2009)**+++**	210	163	**Sound vs. Carious dentin** (Tjäderhane et al., 1998): Present in carious dentin – NA (Vidal et al., 2014): More in carious dentin – NA (Boushell et al., 2011): More in caries-affected tubules (p < 0.05) (Shimada et al., 2009; Gomes-Silva et al., 2017a); NS carious vs. sound (p > 0.05) (Toledano et al., 2010): More in caries-affected – NA (Charadram et al., 2012; Shimada et al., 2009): More in carious dentin - carious vs. sound (p < 0.05) **Lesion depth and location** (Ballal et al., 2017) NS Location (p > 0.05) (Boushell et al., 2011) More in outer sound dentin (p < 0.05) (Shimada et al., 2009): NS Location (p > 0.05) (Vidal et al., 2014): More in inner dentin – NA (Shimada et al., 2009; Gomes-Silva et al., 2017a): More in dentin-enamel junction – NA **Root vs. Coronal** (Toledano et al., 2010) No differences – NA **Before vs. after cavity sealing** (Chibinski et al., 2014; Kuhn et al., 2016) NS before vs. after sealing (p > 0.05)	⊕⚪⚪⚪ Very low
MMP-8	(Tjäderhane et al., 1998) **++** (Chibinski et al., 2014) **++** (Shimada et al., 2009)**++** (Kuhn et al., 2016) **++**	90	90	**Sound vs. Carious dentin/Lesion depth and location** (Tjäderhane et al., 1998): Present in carious dentin – NA (Shimada et al., 2009): More in sound dentin, but also increased in outer carious dentin (p < 0.05) **Before vs. after cavity sealing** (Chibinski et al., 2014), (Kuhn et al., 2016): More in baseline (carious dentin) (p < 0.05)	⊕⚪⚪⚪ Very low
MMP-9	(Tjäderhane et al., 1998) **++** (Vidal et al., 2014) **+** (Chibinski et al., 2014) **++** (Ballal et al., 2017)**++** (Gomes-Silva et al., 2017a) **++** (Shimada et al., 2009) **++** (Kuhn et al., 2016)**++**	164	128	***Sound vs. Carious dentin/Lesion depth and location*** (Tjäderhane et al., 1998): Present in carious dentin – NA (Vidal et al., 2014): More in carious dentin – NA (Shimada et al., 2009): More in sound dentin, but also increased in outer carious dentin (p < 0.05) (Ballal et al., 2017): More in deep than shallow carious tissue (p < 0.05) (Gomes-Silva et al., 2017a): Positive in carious dentin – NA ***Before vs. after cavity sealing*** (Chibinski et al., 2014; Kuhn et al., 2016): NS before vs. after sealing (p > 0.05)	⊕⚪⚪⚪ Very low
MMP-13	(Loreto et al., 2014) **++** (Yeon Lee et al., 2013) **++**	17	10	***Sound vs. Carious dentin*** (Loreto et al., 2014): More in coronal caries group (p < 0.05) ***Root vs. Coronal*** (Yeon Lee et al., 2013): More in root lesions – NA	⊕⚪⚪⚪ Very low
MMP-20	(Gomes-Silva et al., 2017b) **++** (Shimada et al., 2009) **++**	41	41	**Irradiated vs. non irradiated** (Gomes-Silva et al., 2017b): NS **Lesion depth and location** (Shimada et al., 2009): Significantly lower in outer dentin when compared to inner.	⊕⚪⚪⚪ Very low ⊕⊕⊕⚪ Moderate
CT-B	(Vidal et al., 2014) **+** (Nascimento et al., 2011) **++**	13	9	**Sound vs. Carious dentin** (Vidal et al., 2014), (Nascimento et al., 2011): Significantly higher in in caries than sound dentin (p < 0.05)	⊕⚪⚪⚪ Very low

MMP, Metaloproteinase.

CT-B, Cathepsin B.

NA, statistics not available.

NS, non-significant.

**+** = low methodological quality; **++** moderate methodological quality; **+++** high methodological quality.

**Table 3:** Characteristics and methodological quality of individual studies evaluating bacterial collagenases according to collagenase activity or gene expression (N=4).

Author, year, country	Sample location	Type of collagenase	Method used to identify/quantify collagenases	Main results	Methodological quality
Collagenase activity					
(Bello Arroyo, 2016); Spain	Coronal (n=3 dentin samples)	Non-characterized bacterial collagenases	Collagenase assay testing isolates	No collagenolytic activity (low concentration of collagen in the culture media used).	**+++**
(Hashimoto et al., 2011); Japan	Root (n=6 dentin samples)	Non-characterized bacterial collagenases	SDS-PAGE testing isolates for collagen degradation	Protein-degrading bacteria (the ones forming clear zones around their colonies on the FAA-skim milk plates) **isolated from biofilm on root caries lesions were capable of degrading collagen** (*Prevotella, Actinobaculum* and *Propionibacterium* were predominant within this group); Protein-coagulating bacteria did not degrade collagen (the ones forming whitish-coagulating zones around their colonies on the FAA-skim milk plates), but produced enough organic acids to denature proteins; The proportion of protein-degrading in root caries was 7%.	**++**
Collagenases gene expression					
(N. Damé-Teixeira et al., 2018); Brazil	Root; Caries (n=9) vs. sound (n=10)	Most peptidase U32 Collagenase-like protease, PrtC family	Omics	42 bacterial collagenolytic proteases with significant differential expression, from which **18 were superexpressed in root caries**	**+++**
(Simon-Soro et al., 2013); Spain	Coronal; Dentin (n=3) vs. enamel (n=3)	Non-characterized bacterial collagenases or proteases	Omics	Genes coding for **collagenases and other proteases overrepresented in dentin caries when compared to enamel caries.**	**+++**

++ moderate methodological quality; +++ high methodological quality.

The heterogeneity in the methods and protocols made comparisons across studies particularly difficult. The qualitative synthesis of selected articles will be presented by dividing them into host collagenases and bacterial collagenases.

### 3.1 Host-derived collagenolytic proteases

Data on host-derived proteases were generally reported according to the location within the lesions and the enzymatic activity abundance in the different sites. Two studies applied enzymatic assays, and nine applied immunohistochemistry methods. No studies considered the age of the individuals or other clinical characteristics as confounding factors, except the ones from Gomes-Silva et al. (Gomes-Silva et al., 2017a; Gomes-Silva et al., 2017b), Nascimento et al. (Nascimento et al., 2011), and Charadram et al. (Charadram et al., 2012) (**Table 1**; **Supplementary Appendix 4**).

Ten out of 18 included studies assessed the prevalence of the gelatinase MMP-2, comprising a total of 210 carious samples and 163 sound dentin samples evaluated across all the literature. From those, five evaluated more than one sample per teeth considering the lesion depth (Shimada et al., 2009; Toledano et al., 2010; Chibinski et al., 2014; Vidal et al., 2014) or location (Vidal et al., 2014; Kuhn et al., 2016). This represented the most studied MMP. **Table 2** summarizes of the qualitative, quantitative and certainty of the evidence for MMP-2, MMP-8, MMP-9, MMP-13, MMP-20, cysteine cathepsins (CTs) B and K for different paired comparisons (sound vs. carious dentin; root vs. coronal caries; lesion depth and location - outer vs. inner dentin; irradiated vs. non-irradiated), as detailed below.

#### 3.1.1 Sound vs. carious

MMP-2 may be increased in carious dentin when compared to sound dentin, but the evidence is very uncertain. Two out of seven studies found no significant differences between sound and carious tissues (Shimada et al., 2009; Boushell et al., 2011) or regarding the level of caries severity (Boushell et al., 2011). However, other five studies showed significant higher presence/activity of MMP-2 in carious dentin when compared to sound ones, suggesting an effect direction (**Table 2**). The low methodological quality of 3 out of 7 studies was the main reason for serious risk of bias, while the imprecision was serious as the total sample size (N=210 carious and 163 sound) did not reach the Optimal Information Size (OIS) (**Supplementary Appendix 6.1**).

Also, MMP-9, MMP-13 and CT-B may be increased in carious dentin when compared to sound dentin, but the evidence is very uncertain. Due to the conflicting results of the included studies, no effect direction was found so the evidence is very uncertain regarding the MMP-8 presence in carious vs. sound dentin (**Supplementary Appendix 6**). Higher intensity of immunodetection of CTs B and K, and MMPs in general was registered in carious dentin than in sound dentin, though only five samples were analyzed per group (Vidal et al., 2014). The same pattern of increased immunoexpression was also observed regarding CT-B (Nascimento et al., 2011) and MMP-13 (Loreto et al., 2014). The opposite direction was observed by Shimada et al. (Shimada et al., 2009); they found a significant decrease of MMP-8 and MMP-9 in the inner carious dentin compared to sound dentin, and, in their study, the MMP-20 was the highest prevalent in sound dentin (Shimada et al., 2009).

#### 3.1.2 Coronal vs. root dentin caries

For this paired comparison, two host-derived collagenases were studied across the literature. There is no difference in MMP-2 activity in root vs. coronal carious dentin. Immunofluorescence results from one study (Toledano et al., 2010) showed that MMP-2 was present in both coronal and root dentin in all specimens of extracted teeth (sound and carious). MMP-13 may be increased in root caries when compared to coronal, but the evidence is very uncertain (**Supplementary Appendix 6.1, .4**). It has to be noted that no MMP-13 activity was observed in the crown region by western blot methods, suggesting its exclusive presence in root caries lesions (Yeon Lee et al., 2013). However, another study showed an increase in coronal caries when compared to sound dentin (Loreto et al., 2014).

#### 3.1.3 Lesion depth and location

**Figure 2:**
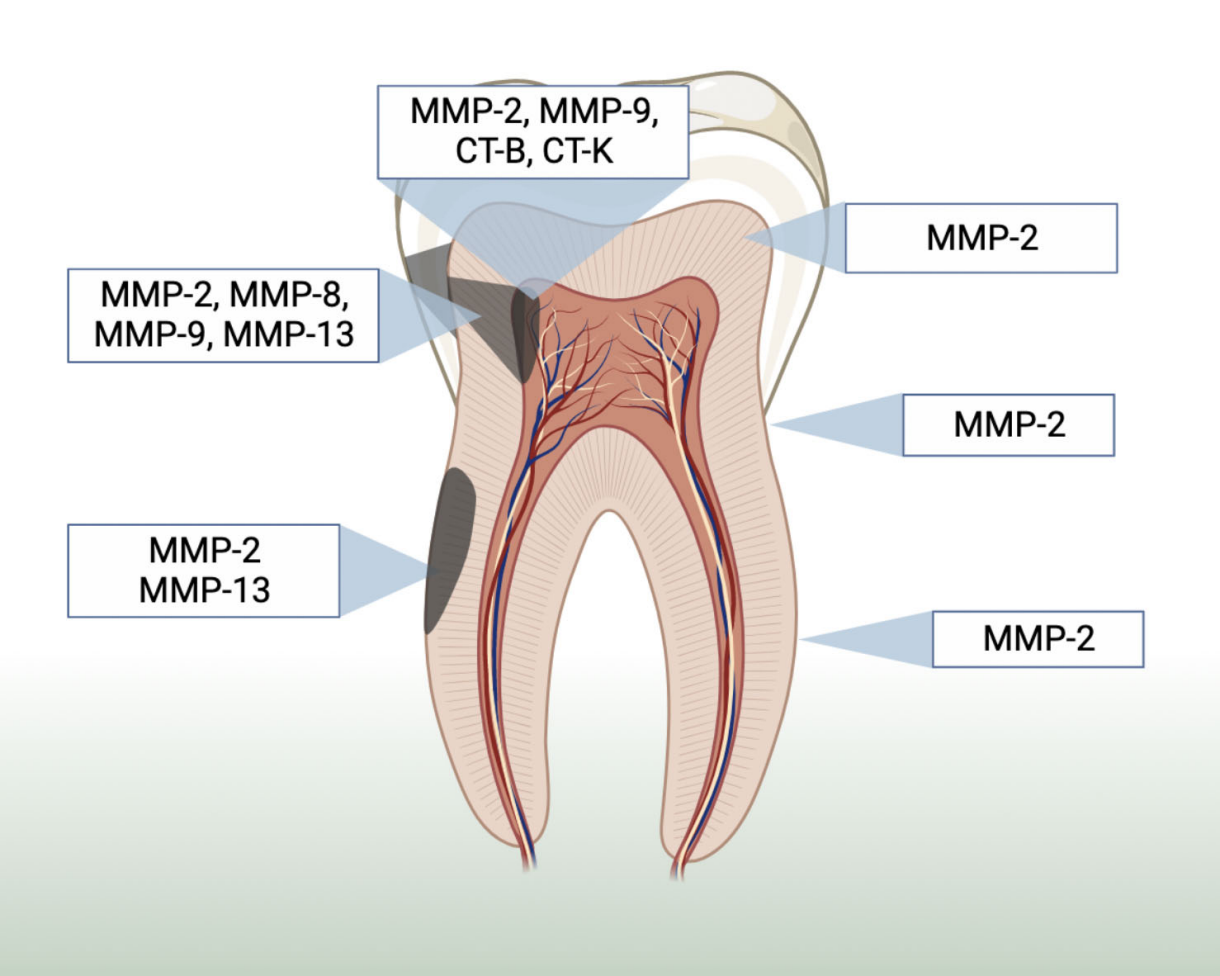
Summary of the location of host collagenases MMPs-2, 8, 9 and 13, as well as cysteine ​​cathepsins B and K based on the included studies.

**Figure 2** illustrates a summary of the location of host collagenases MMPs according to the included studies. Some MMPs activity and/or presence were more significant the closer the lesion was to the pulp and in root dentin (when compared to coronal dentin) (Toledano et al., 2010; Yeon Lee et al., 2013; Vidal et al., 2014; Ballal et al., 2017). Eight studies used immunohistochemistry assays to evaluate the presence and location of host collagenases in carious samples (Shimada et al., 2009; Boushell et al., 2011; Nascimento et al., 2011; Vidal et al., 2014; Kuhn et al., 2016; Ballal et al., 2017). Although some of them described an increase in the collagenolytic proteases expression and activity according to the closer proximity to the pulp tissue, they evaluated different types of collagenases making the cross-study comparison intricate (Toledano et al., 2010; Nascimento et al., 2011; Yeon Lee et al., 2013; Vidal et al., 2014; Ballal et al., 2017).

While a study showed statistically significant increase in the level of MMP-2 immunoreactivity in tubules affected by caries across all samples (Boushell et al., 2011), other studies showed that MMP-2 was ubiquitous (present in sound and carious, root and coronal dentin) and varied considerably in different regions of the dentin (Toledano et al., 2010; Kuhn et al., 2016). Consequently, the evidence is very uncertain about the MMP-2 location in dentin, as no effect direction was found (**Supplementary Appendix 6.1**). The same pattern was observed regarding MMP-8 and 9, i.e. generalized distribution in the intertubular dentin, with no statistical differences when considering their location (Kuhn et al., 2016). The evidence is very uncertain about the MMP-9 expression in different locations and lesion depth, as studies showed opposite effect directions. However, MMP-20 probably reduces in carious outer dentin when compared to carious inner dentin (moderate evidence). The MMP-20 expression was intense along the dentin-enamel junction (Gomes-Silva et al., 2017b).

#### 3.1.4 Before and after cavity sealing

Although the evidence is very uncertain, there is no difference of MMP-2 and MMP-9 expression/activity before and after cavity sealing, however, MMP-8 may be increased in the dentin before when compared to dentin after cavity sealing. The imprecision was the reason for downgrading, as the OIS was not reached (**Supplementary Appendix 6**). The intensity of immunostaining of MMP-2 and MMP-9 was similar between dentin samples collected before and after restoration with glass ionomer cement (after 60 days), being the MMP-9 distributed in the intertubular dentin for both periods in all samples (Kuhn et al., 2016). The same study showed that MMP-8 was observed in all samples at baseline, but significantly reduced after 60 days of sealing (Kuhn et al., 2016). Chibinski et al., that evaluated the expression of the same proteases, in addition to type I collagen and bone sialoprotein (BSP) in infected dentin after sealing with glass ionomer, revealed a significant decrease of MMP-8 in carious dentin after cavity sealing (Chibinski et al., 2014). MMP-2 and 9 were more concentrated around the dentinal tubules.

#### 3.1.5 Irradiated vs. non-irradiated dentin

There is no difference about the MMP-20 expression in irradiated vs. non-irradiated carious dentin, but the evidence is very uncertain (**Supplementary Appendix 6.5**). Gomes-Silva et al. (Gomes-Silva et al., 2017a; Gomes-Silva et al., 2017b) evaluated the expression and activity of MMP-2 e MMP-20 in dentin-enamel junction and in the dentin-pulp complex of irradiated subjects, with no significant difference in either activity or gelatinolytic expression between the irradiated and non-irradiated groups.

### 3.2 Bacterial-derived collagenolytic proteases

While the available studies on this topic are relatively scarce, they do suggest a potential role for bacterial-derived collagenases in dentinal and root caries (**Table 3**; **Supplementary Appendix 5**). To facilitate a comprehensive understanding, the knowledge synthesis will be categorized based on the methodology employed. It is important to note that due to the limited number and diversity of these studies, the application of the GRADE system was not feasible, emphasizing the pressing need for additional research in this area.

#### 3.2.1 Collagenase enzymatic assays

Two studies assayed samples from nine subjects for microbial collagenase using ELISA and SDS-PAGE methods (Hashimoto et al., 2011; Bello Arroyo, 2016). The results are inconclusive: while one identified bacterial isolates capable of degrading protein in root lesions (Hashimoto et al., 2011), another has failed to demonstrate collagenolytic activity in the biofilm of dentinal lesions (Bello Arroyo, 2016). This fact, however, can be explained by the small amount of collagen present in the culture medium used or the small sample size (only 3 subjects) (Bello Arroyo, 2016).

Some protein-degrading bacteria were detected in the biofilm of root carious lesions, such as *Actinobaculum*, *Prevotella* and *Propionibacterium*. For this study, the clinical characteristics of six individuals were evaluated, such as age, gender, plaque and counts of colony unity forming. However, it is important to emphasize that the sample number was small to be able to infer the importance of this result (Hashimoto et al., 2011). These bacteria were capable of degrading collagen. The proportions of protein-degrading and protein-clotting bacteria (acid-production capable of protein denaturation) in root caries lesions, supragingival biofilm from sound sites and periodontitis subgingival biofilm were 7 and 33%, 0 and 26% and 17 and 40% of the microbiota, respectively (Hashimoto et al., 2011).

#### 3.2.2 Omics

Two studies found the gene expression of bacterial collagenases using omics data, both presenting functional analysis and showing significant expression of genes coding for collagenolytic proteases in coronal (Simon-Soro et al., 2013) and root caries (Damé-Teixeira et al., 2018). A few bacterial collagenolytic proteases had high gene expression​ values in root surfaces biofilms (SMU_761 and SMU_759 from *S. mutans* and RS05935 from *V. parvula*), while others were overexpressed on root caries (Log2 fold change > 8) when compared to sound root surfaces biofilms comprised *P. alactolyticus* [HMPREF0721_RS02020], *S. inopinata* JCM 12537 [SCIP_RS02440], *P. alactolyticus* [HMPREF0721_RS04640] and *O. uli* DSM7084 [OLSU_RS02990] (Damé-Teixeira et al., 2018).

## 4 Discussion

The role of host and bacterial collagenolytic proteases in distinct mechanisms involved in the development of carious lesions has not been fully elucidated yet. Although some research has been conducted linking dentinal collagen degradation and caries progression with host proteases activation, most studies corresponded to *in vitro* and *in situ* designs. In this systematic review, we found 18 studies evaluating *ex vivo* samples from clinical studies. A high heterogeneity precluded quantitative comparisons between studies, however, trends in the direction of the effect were observed for some host-derived collagenolytic proteases in carious dentin. Genes coding for bacterial collagenolytic proteases and protein-degrading bacteria were detected in coronal and root dentin carious lesions.

The GRADE approach confirmed a very low certainty of the body of evidence for almost all analyzed comparisons. The reasons for each downgrade and documentation of all assessments of the certainty of the body of evidence are available in **Supplementary Appendix 6**. However, it is important to emphasize that the GRADE approach, when used for systematic reviews of association, always consider observational studies as low certainty of evidence, which means that any downgrading will result in a very low certainty of evidence. Main reasons for other downgrading were methodological issues (risk of bias or methodological quality) of included studies, imprecision and inconsistency. The GRADE rule of thumb for imprecision considers an Optimal Information Size (OIS) of 800 samples (considering tests and controls) for continuous variables. For this reason and considering the specificities of this kind of study (*ex vivo*), we preferred to calculate the OIS whenever it was possible. However, for most comparisons it was not possible, then a conservative attitude was accomplished by following the GRADE recommendation.

Inconsistency was the reason for few downgrades, as studies had conflicting results and no clear effect direction was found for the presence of MMP-2 (lesion depth and location) and MMP-9 (sound vs. carious dentin; lesion depth and location). These conflicting results are potentially associated with differences in the sensitivity of the assays measuring the collagenolytic activity. Other potential explanation for discrepancies may rely on the absence of information about the clinical characteristics of the samples’ donors, such as sex, age, diet, salivary flow, caries activity, etc. as studies were mostly descriptive in nature and not always explore the potential role of proteases in the carious process according to other clinical variables.

This systematic review had a comprehensive search in several databases and grey literature, without restriction on language or time, with a reduced chance of publication bias. For this reason, there was no downgrade due to publication bias in the GRADE system. Also, indirectness resulted in no downgrading, since all included studies answered one or more review questions, and fulfilled all the eligibility criteria. Whether there is no downgrading due to the main GRADE domain, it is possible to consider upgrading the evidence certainty for some comparison, in the presence of large magnitude of effect, dose-response gradient or if plausible confounding can increase confidence in estimated effects (Guyatt et al., 2011). In this review, the referred large magnitude was only observed for MMP-20 comparison in carious outer dentin vs. carious inner dentin, which resulted in moderate certainty of evidence for higher expression in carious outer dentin.

It has been suggested that carious lesions in dentin development involve two stages (Nyvad and Fejerskov, 1990). Initially, there is a demineralization phase, which maintains the characteristic cross-banding of collagen fibers (Nyvad and Fejerskov, 1990; Deyhle et al., 2011). Subsequently, in a second stage, dentinal undergoes degradation by proteolytic enzymes, leading to alterations in the structural characteristics of collagen fibers (Takahashi and Nyvad, 2016). Tjäderhane et al. (Tjäderhane et al., 2015) have suggested that collagen bands could also be degraded during the demineralization phase by host-derived collagenolytic proteases activation. Although the precise physiological functions of these enzymes in dentin are not yet well understood, they may be involved in the formation of peritubular and tertiary dentin, as well as in the release of dentinal growth factors. These growth factors, in turn, could regulate defensive responses in the pulp (Wahlgren et al., 2002; Hannas et al., 2007; Mazzoni et al., 2012; Muromachi et al., 2012; Charadram et al., 2013; Tjäderhane et al., 2013; Tjäderhane et al., 2015). Some of these enzymes have been shown to be capable and sufficient to degrade demineralized dentin *in vitro* (Tjäderhane et al., 1998; Tezvergil-Mutluay et al., 2010). In this context, the gelatinase MMP-2 can be involved in the spread of caries. More attention should be given to MMP-9, MMP-13, and CT-B in further studies as they may also be increased in carious dentin.

The controversy about scientific evidence for bacterial collagenases activity in dentin has raged unabated. Some reports have indicated that the predominant microorganisms may lack the capability to degrade the collagen matrix in cavitated caries lesions (Kawasaki and Featherstone, 1997; Tjäderhane et al., 1998), and that cariogenic bacteria could not completely degrade the organic matrix of dentin after demineralization (Lynch and Ten Cate, 2006). Collagen ordinarily adopts a robust triple helical structure, endowing it with strength and stability. Nevertheless, as previously discussed, exposure to an acidic pH can disrupt specific cross-links responsible for maintaining this triple helical configuration in deeper layers of the cementum, where collagen fibers alternate with and without crystals, and in where a predominant gram-positive bacterial invasion can be observed (Nyvad and Fejerskov, 1990). A potential role was previously discarded by investigating 32 streptococci and lactobacilli isolates, from which only one colony showed faint gelatinolytic activity in enzymography *ex vivo* (Tjäderhane et al., 1998). However, these studies analyzed isolated microorganisms, disregarding the biofilm as a whole, the presence of other microorganisms and their complex metabolic interaction. On the other hand, recent advances in molecular methods (including NGS technologies) suggest a potential role of these collagenases in caries, showing a massive presence of proteolytic bacteria and the overexpression of genes encoding collagenases and other proteases (N Hashimoto et al., 2011; Simon-Soro et al., 2013; Damé-Teixeira et al., 2018), although it is important to take into account that gene expression does not mean enzymatic activity. However, some well-known oral bacteria involved in oral diseases produce their own collagenases that can be capable of breaking down the dentinal collagen (Harrington, 1996). Interestingly, a recent study showed that the bacterial composition of root caries lesions located under the gingival margin is likely to have periodontal pathobionts: *Porphyromonas, Selenomonas, Filifactor, Peptococcus* and *Tannerela* inhabit root caries lesions that extend beyond the gingival margin (Takenaka et al., 2021). This suggests that the microbiome in root caries lesions expanding across the gingival margin would show an increase in proteolytic bacterial diversity. Furthermore, the recent integrated ecological hypothesis for caries and periodontitis (Nyvad and Takahashi, 2020) points to a common risk factor for both diseases, which are originated in the dynamic stability stage and emerged in response to nutritional unbalance in the microbiota.

The difficulty of comparing the findings across all included studies due to high heterogeneity is a limitation of this study. In addition, few included studies were classified as “low” methodological quality, which reduces the strength of the scientific evidence. These findings reinforce the need for further research aiming to identify and characterize both host and bacterial collagenolytic proteases. In the long term, unravelling the role of proteolytic bacteria in the degradation of the dentin matrix could open the way for new therapeutic measures in the treatment of dental caries.

In conclusion, although the evidence was very uncertain, it was possible to assume that 1) MMP-2, MMP-9, MMP-13, and CT-B may be increased in carious dentin when compared to sound dentin; 2) there is no difference in MMP-2 presence, while MMP-13 may be increased in root when compared to coronal carious dentin; 3) there is no difference of MMP-2 and MMP-9 expression/activity before and after cavity sealing; 4) MMP-8 may be increased in the dentin before cavity sealing when compared to dentin after cavity sealing; 5) there is no difference about the MMP-20 expression in irradiated vs. non-irradiated carious dentin. MMP-2 was present in almost all samples studied across the literature, and no effect direction was found in its presence regarding lesion depth and location. MMP-20 probably reduces in carious outer dentin when compared to carious inner dentin (moderate certainty of the evidence). It is crucial to underscore the need for standardizing enzyme assays to enhance the detection of proteolytic activity. Furthermore, researchers should consider employing larger sample sizes compared to those utilized in the studies featured in this article. This will help improve the accuracy and reliability of collagenase detection methods. For bacterial proteases, there is controversy over the scientific evidence of their activity in carious lesions, in addition to a significantly smaller number of studies focused on microbial proteolysis. However, genes encoding bacterial collagenolytic proteases and protein-degrading bacteria have already been seen with considerable prevalence in carious lesions and can represent a potential target for biofilm modulation.

## Author’s note 

This systematic review was reported according to the Preferred Reporting Items for Systematic Review and Meta-Analysis (PRISMA) checklist (Page et al., 2021). A study protocol was designed and registered at the International Prospective Register of Systematic Review (PROSPERO) database, under the identification number CRD42020213141.

## Author contributions

CB: Conceptualization, Data curation, Formal Analysis, Investigation, Methodology, Writing – original draft, Writing – review & editing. IM: Conceptualization, Data curation, Formal Analysis, Investigation, Methodology, Writing – original draft, Writing – review & editing. JC: Conceptualization, Data curation, Formal Analysis, Investigation, Methodology, Writing – review & editing. CS: Conceptualization, Formal Analysis, Investigation, Methodology, Project administration, Resources, Supervision, Visualization, Writing – review & editing. ND-T: Conceptualization, Funding acquisition, Investigation, Project administration, Resources, Supervision, Validation, Visualization, Writing – review & editing.

## References

[B1] Ballal V. Rao S. Bagheri A. Bhat V. Attin T. Zehnder M. (2017). MMP-9 in dentinal fluid correlates with caries lesion depth. Caries Res. 51 (5), 460–465. doi: 10.1159/000479040 28848154

[B2] Bello Arroyo E. (2016) caracterizatción de microrganismos aislados de caries de dentina. Available at: http://hdl.handle.net/10251/62085.

[B3] Boushell L. W. Nagaoka H. Yamauchi M. (2011). Increased matrix metalloproteinase-2 and bone sialoprotein response to human coronal caries. Caries Res. 45 (5), 453–459. doi: 10.1159/000330601 21876355 PMC3182042

[B4] Campbell M. McKenzie J. E. Sowden A. Katikireddi S. V. Brennan S. E. Ellis S.. (2020). Synthesis without meta-analysis (SWiM) in systematic reviews: reporting guideline. Bmj 368, l6890. doi: 10.1136/bmj.l6890 31948937 PMC7190266

[B5] Charadram N. Austin C. Trimby P. Simonian M. Swain M. V. Hunter N. (2013). Structural analysis of reactionary dentin formed in response to polymicrobial invasion. J. Struct. Biol. 181 (3), 207–222. doi: 10.1016/j.jsb.2012.12.005 23261402 PMC3578079

[B6] Charadram N. Farahani R. M. Harty D. Rathsam C. Swain M. V. Hunter N. (2012). Regulation of reactionary dentin formation by odontoblasts in response to polymicrobial invasion of dentin matrix. Bone 50 (1), 265–275. doi: 10.1016/j.bone.2011.10.031 22079283 PMC3246533

[B7] Chaussain-Miller C. Fioretti F. Goldberg M. Menashi S. (2006). The role of matrix metalloproteinases (MMPs) in human caries. J. Dent. Res. 85 (1), 22–32. doi: 10.1177/154405910608500104 16373676

[B8] Chibinski A. C. Gomes J. R. Camargo K. Reis A. Wambier D. S. (2014). Bone sialoprotein, matrix metalloproteinases and type I collagen expression after sealing infected caries dentin in primary teeth. Caries Res. 48 (4), 312–319. doi: 10.1159/000355302 24556583

[B9] Dame-Teixeira N. de Lima A. K. A. Do T. Stefani C. M. (2021). Meta-analysis using NGS data: the veillonella species in dental caries. Front. Oral. Health 2. doi: 10.3389/froh.2021.770917 PMC8757819 35048071

[B10] Damé-Teixeira N. Parolo C. C. F. Maltz M. Rup A. G. Devine D. A. Do T. (2018). Gene expression of bacterial collagenolytic proteases in root caries. J. Oral. Microbiol. 10 (1), 1424475. doi: 10.1080/20002297.2018.1424475 34394852 PMC5774410

[B11] Dayan D. Binderman I. Mechanic G. L. (1983). A preliminary study of activation of collagenase in carious human dentine matrix. Arch. Oral. Biol. 28 (2), 185–187. doi: 10.1016/0003-9969(83)90126-7 6307237

[B12] Deyhle H. Bunk O. Muller B. (2011). Nanostructure of healthy and caries-affected human teeth. Nanomedicine 7 (6), 694–701. doi: 10.1016/j.nano.2011.09.005 21945898

[B13] Gomes-Silva W. Prado-Ribeiro A. C. Brandão T. B. Morais-Faria K. de Castro Junior G. Mak M. P.. (2017b). Postradiation matrix metalloproteinase-20 expression and its impact on dental micromorphology and radiation-related caries. Caries Res. 51 (3), 216–224. doi: 10.1159/000457806 28359051

[B14] Gomes-Silva W. Prado Ribeiro A. C. de Castro Junior G. Salvajoli J. V. Rangel Palmier N. Lopes M. A.. (2017a). Head and neck radiotherapy does not increase gelatinase (metalloproteinase-2 and -9) expression or activity in teeth irradiated in vivo. Oral. Surg. Oral. Med. Oral. Pathol. Oral. Radiol. 124 (2), 175–182. doi: 10.1016/j.oooo.2017.04.009 28602264

[B15] Guyatt G. H. Oxman A. D. Sultan S. Glasziou P. Akl E. A. Alonso-Coello P. (2011). GRADE guidelines: 9. Rating up the quality of evidence. J Clin Epidemiol 64 (12), 1311–1316. 21802902 10.1016/j.jclinepi.2011.06.004

[B16] Griffin S. O. Griffin P. M. Swann J. L. Zlobin N. (2004). Estimating rates of new root caries in older adults. J. Dent. Res. 83 (8), 634–638. doi: 10.1177/154405910408300810 15271973

[B17] Hannas A. R. Pereira J. C. Granjeiro J. M. Tjäderhane L. (2007). The role of matrix metalloproteinases in the oral environment. Acta Odontol Scand. 65 (1), 1–13. doi: 10.1080/00016350600963640 17354089

[B18] Harrington D. J. (1996). Bacterial collagenases and collagen-degrading enzymes and their potential role in human disease. Infect. Immun. 64 (6), 1885–1891. doi: 10.1128/iai.64.6.1885-1891.1996 8675283 PMC174012

[B19] Hashimoto K. Sato T. Shimauchi H. Takahashi N. (2011). Profiling of dental plaque microflora on root caries lesions and the protein-denaturing activity of these bacteria. Am. J. Dent. 24 (5), 295–299. 22165457

[B20] Kassab M. M. Cohen R. E. (2003). The etiology and prevalence of gingival recession. J. Am. Dent. Assoc. 134 (2), 220–225. doi: 10.14219/jada.archive.2003.0137 12636127

[B21] Kassebaum N. J. Bernabé E. Dahiya M. Bhandari B. Murray C. J. Marcenes W. (2015). Global burden of untreated caries: a systematic review and metaregression. J. Dent. Res. 94 (5), 650–658. doi: 10.1177/0022034515573272 25740856

[B22] Kawasaki K. Featherstone J. D. (1997). Effects of collagenase on root demineralization. J. Dent. Res. 76 (1), 588–595. doi: 10.1177/00220345970760011001 9042082

[B23] Kuhn E. Reis A. Campagnoli E. B. Chibinski A. C. Carrilho M. R. Wambier D. S. (2016). Effect of sealing infected dentin with glass ionomer cement on the abundance and localization of MMP-2, MMP-8, and MMP-9 in young permanent molars in *vivo* . Int. J. Paediatr. Dent. 26 (2), 125–133. doi: 10.1111/ipd.12167 25967636

[B24] Loreto C. Galanti C. Musumeci G. Rusu M. C. Leonardi R. (2014). Immunohistochemical analysis of matrix metalloproteinase-13 in human caries dentin. Eur. J. Histochem 58 (1), 2318. doi: 10.4081/ejh.2014.2318 24704999 PMC3980212

[B25] Lynch R. J. Ten Cate J. M. (2006). The effect of lesion characteristics at baseline on subsequent de- and remineralisation behaviour. Caries Res. 40 (6), 530–535. doi: 10.1159/000095653 17063025

[B26] Mazzoni A. Nascimento F. D. Carrilho M. Tersariol I. Papa V. Tjäderhane L.. (2012). MMP activity in the hybrid layer detected with in *situ* zymography. J. Dent. Res. 91 (5), 467–472. doi: 10.1177/0022034512439210 22354448 PMC3327728

[B27] Murad M. H. Mustafa R. A. Schünemann H. J. Sultan S. Santesso N. (2017). Rating the certainty in evidence in the absence of a single estimate of effect. Evid Based Med. 22 (3), 85–87. doi: 10.1136/ebmed-2017-110668 28320705 PMC5502230

[B28] Muromachi K. Kamio N. Matsumoto T. Matsushima K. (2012). Role of CTGF/CCN2 in reparative dentinogenesis in human dental pulp. J. Oral. Sci. 54 (1), 47–54. doi: 10.2334/josnusd.54.47 22466886

[B29] Nascimento F. D. Minciotti C. L. Geraldeli S. Carrilho M. R. Pashley D. H. Tay F. R.. (2011). Cysteine cathepsins in human carious dentin. J. Dent. Res. 90 (4), 506–511. doi: 10.1177/0022034510391906 21248362 PMC3144127

[B30] Nyvad B. Fejerskov O. (1990). An ultrastructural study of bacterial invasion and tissue breakdown in human experimental root-surface caries. J. Dent. Res. 69 (5), 1118–1125. doi: 10.1177/00220345900690050101 2335644

[B31] Nyvad B. Takahashi N. (2020). Integrated hypothesis of dental caries and periodontal diseases. J. Oral. Microbiol. 12 (1), 1710953. doi: 10.1080/20002297.2019.1710953 32002131 PMC6968559

[B32] Page M. J. McKenzie J. E. Bossuyt P. M. Boutron I. Hoffmann T. C. Mulrow C. D.. (2021). The PRISMA 2020 statement: an updated guideline for reporting systematic reviews. Bmj 372, n71. doi: 10.1136/bmj.n71 33782057 PMC8005924

[B33] Santesso N. Glenton C. Dahm P. Garner P. Akl E. A. Alper B.. (2020). GRADE guidelines 26: informative statements to communicate the findings of systematic reviews of interventions. J. Clin. Epidemiol. 119, 126–135. doi: 10.1016/j.jclinepi.2019.10.014 31711912

[B34] Severo Alves L. Dam-Teixeira N. Susin C. Maltz M. (2013). Association among quality of life, dental caries treatment and intraoral distribution in 12-year-old South Brazilian schoolchildren. Community Dent. Oral. Epidemiol. 41 (1), 22–29. doi: 10.1111/j.1600-0528.2012.00707.x 22882480

[B35] Shimada Y. Ichinose S. Sadr A. Burrow M. F. Tagami J. (2009). Localization of matrix metalloproteinases (MMPs-2, 8, 9 and 20) in normal and carious dentine. Aust. Dent. J. 54 (4), 347–354. doi: 10.1111/j.1834-7819.2009.01161.x 20415934

[B36] Simon-Soro A. Belda-Ferre P. Cabrera-Rubio R. Alcaraz L. D. Mira A. (2013). A tissue-dependent hypothesis of dental caries. Caries Res. 47 (6), 591–600. doi: 10.1159/000351663 24080530

[B37] Takahashi N. Nyvad B. (2016). Ecological hypothesis of dentin and root caries. Caries Res. 50 (4), 422–431. doi: 10.1159/000447309 27458979

[B38] Takenaka S. Edanami N. Komatsu Y. Nagata R. Naksagoon T. Sotozono M.. (2021). Periodontal pathogens inhabit root caries lesions extending beyond the gingival margin: A next-generation sequencing analysis. Microorganisms 9 (11). doi: 10.3390/microorganisms9112349 PMC8617989 34835473

[B39] Tezvergil-Mutluay A. Agee K. A. Hoshika T. Carrilho M. Breschi L. Tjäderhane L.. (2010). The requirement of zinc and calcium ions for functional MMP activity in demineralized dentin matrices. Dent. Mater 26 (11), 1059–1067. doi: 10.1016/j.dental.2010.07.006 20688380 PMC2948575

[B40] Tjäderhane L. Buzalaf M. A. Carrilho M. Chaussain C. (2015). Matrix metalloproteinases and other matrix proteinases in relation to cariology: the era of 'dentin degradomics'. Caries Res. 49 (3), 193–208. doi: 10.1159/000363582 25661522

[B41] Tjäderhane L. Larjava H. Sorsa T. Uitto V. J. Larmas M. Salo T. (1998). The activation and function of host matrix metalloproteinases in dentin matrix breakdown in caries lesions. J. Dent. Res. 77 (8), 1622–1629. doi: 10.1177/00220345980770081001 9719036

[B42] Tjäderhane L. Nascimento F. D. Breschi L. Mazzoni A. Tersariol I. L. Geraldeli S.. (2013). Strategies to prevent hydrolytic degradation of the hybrid layer-A review. Dent. Mater 29 (10), 999–1011. doi: 10.1016/j.dental.2013.07.016 23953737 PMC3899917

[B43] Toledano M. Nieto-Aguilar R. Osorio R. Campos A. Osorio E. Tay F. R.. (2010). Differential expression of matrix metalloproteinase-2 in human coronal and radicular sound and carious dentine. J. Dent. 38 (8), 635–640. doi: 10.1016/j.jdent.2010.05.001 20452393

[B44] Vidal C. M. Tjäderhane L. Scaffa P. M. Tersariol I. L. Pashley D. Nader H. B.. (2014). Abundance of MMPs and cysteine cathepsins in caries-affected dentin. J. Dent. Res. 93 (3), 269–274. doi: 10.1177/0022034513516979 24356440

[B45] Wahlgren J. Salo T. Teronen O. Luoto H. Sorsa T. Tjäderhane L. (2002). Matrix metalloproteinase-8 (MMP-8) in pulpal and periapical inflammation and periapical root-canal exudates. Int. Endod. J. 35 (11), 897–904. doi: 10.1046/j.1365-2591.2002.00587.x 12453017

[B46] Yeon Lee T. Jung Jin E. Choi B. (2013). MMP-13 expression in coronal and radicular dentin according to caries progression -a pilot study. Tissue Eng. Regenerative Med. 10, 317–321. doi: 10.1007/s13770-013-1095-8

