# Supplementary material for: Presence of host and bacterial-derived collagenolytic proteases in carious dentin: a systematic review of ex vivo studies
Source: Front Cell Infect Microbiol. 2023 Oct 31;13:1278754. doi: 10.3389/fcimb.2023.1278754 (PMC10644316; doi:10.3389/fcimb.2023.1278754)
Supplement: Supplementary file 1 [file Table_1.docx]

***Presence of host and bacterial-derived collagenolytic proteases in carious dentin: a systematic review of ex vivo studies***

**Supplementary Appendix 1.** Search strategy

| **Database** | **Search strategy** | **Results** |
| --- | --- | --- |
| Cochrane Library | (“Dental Caries“ OR “Root Caries“) in Title Abstract Keyword AND (“Collagenases“ OR “Microbial Collagenase“ OR “metalloproteinase collagen“) in Title Abstract Keyword - (Word variations have been searched) | 4 |
| LILACS | (tw:(Dental Caries OR Dental Decay Caries OR Dental Decay OR Carious Dentin OR Carious Dentins OR Coronal Dentin OR coronal dentin OR Root Caries OR Cervical Caries OR Cervical Cary OR Caries )) AND (tw:(Collagenases OR Collagen-Degrading Enzyme OR Collagen Degrading Enzyme OR Collagenase OR Collagen Peptidase OR Peptidase, Collagen OR Microbial Collagenase OR Collagenase-Like Peptidase OR Collagenase Like Peptidase OR metalloproteinase collagen)) | 2 |
| Google Scholar | (“Dental Caries“ OR “Carious Dentin“ OR “Root Caries“ OR “Caries“) AND (“Collagenases“ OR “Collagen Degrading Enzyme“ OR “Microbial Collagenase“) | 101 |
| PubMed | (Dental Caries [MeSH Terms] OR Dental Decay Caries OR Dental Decay OR Carious Dentin OR Carious Dentins OR Coronal Dentin OR coronal dentin OR Root Caries[MeSH Terms] OR Cervical Caries OR Cervical Cary OR Caries) AND (Collagenases [MeSH Terms] OR Collagen-Degrading Enzyme OR Collagen Degrading Enzyme OR Collagenase OR Collagen Peptidase OR Peptidase, Collagen OR Microbial Collagenase [MeSH Terms] OR Collagenase-Like Peptidase OR Collagenase Like Peptidase OR metalloproteinase collagen) | 215 |
| Web of Science | (Dental Caries OR Dental Decay Caries OR Dental Decay OR Carious Dentin OR Carious Dentins OR Coronal Dentin OR coronal dentin OR Root Caries OR Cervical Caries OR Cervical Cary OR Caries) AND (Collagenases OR Collagen-Degrading Enzyme OR Collagen Degrading Enzyme OR Collagenase OR Collagen Peptidase OR Peptidase, Collagen OR Microbial Collagenase OR Collagenase-Like Peptidase OR Collagenase Like Peptidase OR metalloproteinase collagen) | 176 |
| Embase | (Dental Caries OR Dental Decay Caries OR Dental Decay OR Carious Dentin OR Carious Dentins OR Coronal Dentin OR coronal dentin OR Root Caries OR Cervical Caries OR Cervical Cary OR Caries) AND (Collagenases OR Collagen-Degrading Enzyme OR Collagen Degrading Enzyme OR Collagenase OR Collagen Peptidase OR Peptidase, Collagen OR Microbial Collagenase OR Collagenase-Like Peptidase OR Collagenase Like Peptidase OR metalloproteinase collagen) | 93 |
| Scopus | ( "Collagenases" OR "Collagen Degrading Enzyme" OR "collagenase" OR "Microbial Collagenase" ) AND ( "Dental Caries" OR "Dental Decay Caries" OR "Dental Decay" OR "Carious Dentin" OR "Coronal Dentin" OR "Root Caries" OR "Cervical Caries" OR "Caries" ) | 156 |
| Livivo | (“Dental Caries“ OR “Dental Decay Caries“ OR “Dental Decayv OR “Carious Dentin“ OR “Carious Dentins“ OR “Coronal Dentin“ OR “coronal dentin“ OR “Root Caries“ OR “Cervical Caries“ OR “Cervical Cary“ OR “Caries“) AND (“Collagenases“ OR “Collagen-Degrading Enzyme“ OR “Collagen Degrading Enzyme“ OR “Collagenase“ OR “Collagen Peptidase“ OR “Peptidase, Collagen“ OR “Microbial Collagenase“ OR “Collagenase-Like Peptidase“ OR“ Collagenase Like Peptidase“ OR “metalloproteinase collagen“) | 94 |
| Proquest | (Dental Caries OR Dental Decay Caries OR Dental Decay OR Carious Dentin OR Carious Dentins OR Coronal Dentin OR coronal dentin OR Root Caries OR Cervical Caries OR Cervical Cary OR Caries) AND (Collagenases OR Collagen-Degrading Enzyme OR Collagen Degrading Enzyme OR Collagenase OR Collagen Peptidase OR Peptidase, Collagen OR Microbial Collagenase OR Collagenase-Like Peptidase OR Collagenase Like Peptidase OR metalloproteinase collagen) | 100 |
| OpenGrey | Caries AND collagenases | 0 |

**Supplementary Appendix 2.** Excluded studies and reasons for exclusion (n= 33).

| **Author/Year** | **Reasons for exclusion*** |
| --- | --- |
| Armstrong, 1958 | 2 |
| Ashwini, 2020 | 1 |
| Beltz, 1999 | 2 |
| Damé-Teixeira, 2017 | 3 |
| Dayan, 1983 | 2 |
| Gonçalves, 2021 | 1 |
| Guirado, 2021 | 3 |
| Harrington, 1994 | 1 |
| He, 2005 | 5 |
| Hedenbjork-Lager, 2014 | 3 |
| Hedenbjork-Lager, 2015 | 2 |
| Hedenbjork-Lager, 2016 | 3 |
| Hu, 2019 | 1 |
| Huang, 2021 | 1 |
| Khan, 2021 | 6 |
| Kawasaki, 1997 | 3 |
| Leonardi, 2010 | 6 |
| Niu, 2011 | 1 |
| Schmidt, 2018 | 1 |
| Sulkala, 2004 | 1 |
| Tananure, 2011 | 1 |
| Tannure, 2012 | 1 |
| Van-Strijp, 1992 | 1 |
| Van-Strijp, 1994 | 1 |
| Van-Strijp, 2003 | 1 |
| Vasconcelos, 2019 | 1 |
| Wang, 2018 | 5 |
| Xu, 2011 | 1 |
| Yan, 2014 | 5 |
| Yang, 2006 | 5 |
| Zheng, 2011 | 1 |
| Zheng, 2012 | 1 |
| Zhu, 2012 | 4 |

* 1. Studies performed in sample dental other than coronal and root caries lesions or animal studies (n= 18)

2. In vitro studies (n= 4)

3. Reviews, letters, conference abstracts, personal opinions, book chapter, protocols (n= 5)

4. Articles which the full text could not be found (n=1)

5. Studies written in non-Latin alphabet (n=4)

6. Outcome including other bacterial enzymatic activity that does not collagenases

**References of the excluded articles:**

1. Armstrong, W. G. "Further studies on the action of collagenase on sound and carious human dentin." Journal of Dental Research 37.6 (1958): 1001-1015.
2. Ashwini, Ajay, et al. "Dentin degradonomics–The potential role of salivary MMP-8 in dentin caries." Journal of Clinical and Experimental Dentistry 12.2 (2020): e108.
3. Beltz, R. E., E. C. Herrmann, and H. Nordbö. "Pronase digestion of carious dentin." Caries research 33.6 (1999): 468-472.
4. Damé-Teixeira, Nailê, Clarissa Cavalcanti Fatturi Parolo, and Marisa Maltz. "Specificities of caries on root surface." Root caries: From prevalence to therapy. Vol. 26. Karger Publishers, 2017. 15-25
5. Dayan, D., I. Binderman, and G. L. Mechanic. "A preliminary study of activation of collagenase in carious human dentine matrix." Archives of Oral Biology 28.2 (1983): 185-187.
6. Gonçalves, Rafael Simões, et al. "Two-year randomized clinical trial of different restorative techniques in non-carious cervical lesions and MMP activity in gingival crevicular fluid." Clinical Oral Investigations 26.2 (2022): 1889-1902.
7. Guirado, E., and A. George. "Dentine matrix metalloproteinases as potential mediators of dentine regeneration." Eur. Cells Mater 42 (2021): 392-400.
8. Harrington, Dean J., and Roy RB Russell. "Identification and characterisation of two extracellular proteases of Streptococcus mutans." FEMS microbiology letters 121.2 (1994): 237-241.
9. He, Chang-Li, et al. "The effect of matrix metalloproteinase-1 on root surface dentin matrix: a scanning electron microscope observation." Hua xi kou Qiang yi xue za zhi= Huaxi Kouqiang Yixue Zazhi= West China Journal of Stomatology 23.2 (2005): 113-115.
10. He, Chang-Li, et al. "The effect of matrix metalloproteinase-1 on root surface dentin matrix: a scanning electron microscope observation." Hua xi kou Qiang yi xue za zhi= Huaxi Kouqiang Yixue Zazhi= West China Journal of Stomatology 23.2 (2005): 113-115.
11. Hedenbjörk-Lager, Anders, et al. "Caries correlates strongly with salivary levels of matrix metalloproteinase-8." Caries Research 49.1 (2015): 1-8.
12. Hedenbjörk-Lager, Anders, et al. "Collagen degradation and preservation of MMP-8 activity in human dentine matrix after demineralization." Archives of oral biology 68 (2016): 66-72.
13. Hedenbjörk-Lager, Anders. Dentine caries: acid tolerant microorganisms and aspects on collagen degradation. Diss. Malmö University, Faculty of Odontology, 2014.
14. Hu, Xiao-Pan, et al. "Association of ENAM, TUFT1, MMP13, IL1B, IL10 and IL1RN gene polymorphism and dental caries susceptibility in Chinese children." Journal of International Medical Research 47.4 (2019): 1696-1704.
15. Huang, Bo. Identification and Characterization of Proteolytic Activities from S. mutans that Hydrolyzes Dentinal Collagen Matrix. Diss. University of Toronto (Canada), 2021.
16. Khan, Zaid Majeed, et al. "Differentially expressed salivary proteins in dental caries patients." BioMed Research International 2021 (2021).
17. Kawasaki, K., and J. D. B. Featherstone. "Effects of collagenase on root demineralization." Journal of dental research 76.1 (1997): 588-595.
18. Leonardi, Rosalia, and Carla Loreto. "Immunohistochemical localization of tissue inhibitor of matrix metalloproteinase-1 (TIMP-1) in human carious dentine." Acta histochemica 112.3 (2010): 298-302.
19. Niu, LN1, et al. "Localization of MMP-2, MMP-9, TIMP-1, and TIMP-2 in human coronal dentine." Journal of dentistry 39.8 (2011): 536-542.
20. Schmidt, Jana, et al. "aMMP-8 in correlation to caries and periodontal condition in adolescents—results of the epidemiologic LIFE child study." Clinical Oral Investigations 22.1 (2018): 449-460.
21. Sulkala, Merja, et al. "Matrix metalloproteinase-13 (MMP-13, collagenase-3) is highly expressed in human tooth pulp." Connective tissue research 45.4-5 (2004): 231-237.
22. Tannure, P. N., et al. "MMP13 polymorphism decreases risk for dental caries." Caries research 46.4 (2012): 401-407.
23. Tannure, Patricia Nivoloni, and Erika Calvano Küchler. "Patients with manifest caries lesions have higher levels of salivary matrix metalloproteinase-8 than patients with no caries lesions." Journal of Evidence Based Dental Practice 16.1 (2016): 77-78.
24. Van Strijp, A. J. P., B. Klont, and J. M. Ten Cate. "Solubilization of dentin matrix collagen in situ." Journal of dental research 71.8 (1992): 1498-1502.
25. Van Strijp, A. J. P., et al. "Bacterial colonization and degradation of demineralized dentin matrix in situ." Caries research 28.1 (1994): 21-27.
26. Van-Strijp, A. J. P., et al. "Host-derived proteinases and degradation of dentine collagen in situ." Caries Research 37.1 (2003): 58-65.
27. Vasconcelos, Katia Regina, et al. "MMP13 contributes to dental caries associated with developmental defects of enamel." Caries Research 53.4 (2019): 441-446.
28. Wang, Xiao, and M. Qin. "A preliminary study of saliva matrix metalloproteinases (MMP-2 and MMP-9) in children with caries." Beijing da xue xue bao. Yi xue ban= Journal of Peking University. Health Sciences 50.3 (2018): 527-531.
29. Yan, G. et al. "Relationship between dental caries and salivary proteome by electrospray ionization ion-trap tandem mass spectrometry in children aged 6 to 8 years." West China Journal of Stomatology 32.3 (2014).
30. Yang, D. M., et al. "Effect of host derived matrix metalloproteinase on the degradation of root dentin collagen." Zhonghua kou Qiang yi xue za zhi= Zhonghua Kouqiang Yixue Zazhi= Chinese Journal of Stomatology 41.5 (2006): 275-278.
31. Zheng, X., et al. "Real‐time enzymatic degradation of human dentin collagen fibrils exposed to exogenous collagenase: an AFM study in situ." Journal of microscopy 241.2 (2011): 162-170.
32. Zheng, Xinyu, et al. "AFM study of the effects of collagenase and its inhibitors on dentine collagen fibrils." Journal of Dentistry 40.2 (2012): 163-171.
33. Zhu, Zi-yuan, Tian Zhou, and Bao-wei Zhang. "Characterization and analysis of matrix metalloproteinases 8 and 20 in the human crown and root dentin." Chinese Journal of Tissue Engineering Research 16.24 (2012): 4526.

**Supplementary Appendix 3***.* Quality assessment of the individual included studies (n = 17) using The Joanna Briggs Institute Critical Appraisal Checklist for Cross-sectional Studies. Methodological quality was categorized as high, low or moderate according to critical domain of each question. Criteria adopted to this systematic review for considering a low methodological quality was two “no” or one “no” and one “unclear” in critical domains. High methodological quality was considered when an article got a maximum one “no” answer or two “unclear” answers in non-critical domains. The other ones were classified as moderate.

| **Questions** |  | **Author, Year** | | | | | | | | | | | | | | | | |  |
| --- | --- | --- | --- | --- | --- | --- | --- | --- | --- | --- | --- | --- | --- | --- | --- | --- | --- | --- | --- |
|  | Arroyo and Simón Soro, 2015 | | Boushell et al, 2011 | Charadram et al, 2012 2017 | Damé-Teixeira et al, 2018 | Hashimoto et al, 2011 | Simon-Soro, A. 2013 | Ballal et al, 2017 | Chibinski et al, 2014 | Gomes-Silva et al, 2017a | Gomes-Silva et al, 2017b | Kuhn eta l, 2016 | Lee et al, 2013 | Loreto et al, 2014 | Nascimento, F. D, et.al, 2011 | Shimada, Y, et. al, 2009 | Toledano, M et.al, 2010 | Vidal et al, 2014 | Tjäderhane (1998) |
| **Q1*** | UN | | N | Y | N | Y | Y | Y | Y | Y | Y | Y | UN | UN | Y | UN | N | UN | N |
| **Q2*** | Y | | N | Y | Y | Y | Y | Y | Y | Y | Y | Y | Y | Y | Y | Y | N | UN | Y |
| **Q3*** | Y | | UN | Y | Y | Y | Y | Y | Y | Y | Y | Y | Y | Y | Y | Y | Y | UN | Y |
| **Q4*** | Y | | UN | Y | Y | Y | Y | Y | Y | Y | Y | Y | Y | Y | Y | UN | N | UN | Y |
| **Q5** | N | | NA | NA | NA | N | NA | N | N | N | N | N | N | N | N | NA | N | N | UN |
| **Q6** | N | | NA | NA | NA | N | NA | N | N | N | N | N | N | N | N | NA | N | N | N |
| **Q7** | Y | | Y | Y | Y | Y | Y | Y | Y | Y | Y | Y | Y | Y | Y | Y | Y | Y | Y |
| **Q8** | Y | | Y | Y | Y | Y | Y | Y | Y | Y | Y | Y | NA | Y | Y | Y | NA | Y | NA |
| **MQ** | **H** | | **L** | **H** | **H** | **M** | **H** | **M** | **M** | **M** | **M** | **M** | **M** | **M** | **M** | **M** | **L** | **L** | **M** |

Critical domain (*); methodological quality (MQ); yes (Y), no (N), unclear (U); not applicable (NA); low (L); high (H). Q1 – CRITICAL: Were the criteria for inclusion in the sample clearly defined? Q2 – CRITICAL: Were the study subjects and the setting described in detail? Q3 – CRITICAL: Was the exposure measured in a valid and reliable way? Q4 – CRITICAL: Were objective, standard criteria used for measurement of the condition? Q5 – NON-CRITICAL: Were confounding factors identified? Q6 – NON-CRITICAL: Were strategies to deal with confounding factors stated? Q7– NON-CRITICAL: Were the outcomes measured in a valid and reliable way? Q8 – NON-CRITICAL: Was appropriate statistical analysis used?

**Supplementary Appendix 4.** Data collection from studies evaluating host collagenases, presented according to the method used to collagenase identification/measurement.

| **Collagenase assay** | | | | | | | | | | | | | | | | | |
| --- | --- | --- | --- | --- | --- | --- | --- | --- | --- | --- | --- | --- | --- | --- | --- | --- | --- |
| **Author, year** | **Country** | **N (caries group)** | **N (control group)** | ***Ex vivo* specimen collected** |  | | **Methods** | | **Collagenase assay** | | **Clinical characteristics of the sample** | | | **Type of collagenase** | **Main Conclusions** |  |  |
| Charadram et al, 2012 | Australia | 30 permanent molar teeth carious | 15 sound | Extracted teeth |  | | Realtime qPCR and gelatinase assay | | The protein extracted from reactionary dentin (layer 4) contained specific MMP-2 activity of 19.36 ± 5.13 mU/μg while protein extracted from healthy dentin samples contained specific MMP-2 activity of 3.50 ± 0.12 mU/μg | | Were obtained from male and female patients aged 20 to 35 years | | | MMP – 2 | MMP-2 activity in the Reactionary dentin (layer 4) was significantly higher than the healthy sample. All values depict means ± SD (n=14). * P≤ 0.05; ** P≤ 0.02 |  |  |
| Gomes-Silva et al, 2017b | Brazil | 19 Irradiated carious dentin (15 male and 4 female; mean age: 58-60 years) | 17 Non Irradiated carious dentin | Extracted teeth (demineralized and non-demineralized) |  | | Immunohistochemical;  Gelatinolytic activity assay by zymography | | Gelatinolytic activity analysis of specimens from subgroup 1:  Irradiated x Non irradiated: DEJ 0/7 (0%) x 1/7 (14,2%) p = 0,31;  Caries (I/O) 4/7 (57,1%) x 4/7 (57,1%); Caries (C) 5/7 (71,4%) x 2/7 (28,5%);  Dentinal tubules 4/7 (57,1%) x 5/7 (71,4%);  Pre-dentin 1/7 (14,2%) x 2/7 (28,5%);  Tertiary dentin 2/4 (50%) x 1/4 (25%)  (I = incisal, O = occlusal, C = cervical) | | Erupted teeth (n = 36) from HNC (Head and Neck Cancer) patients.  19 post-HNRT(post Head and Neck Treatment) specimens. In subgroup 1 ten patients were male and 1 patient was female, and in subgroup 2 five patients were male and 3 were female. The mean age was 58 years (range 36–74) and 60 years (range 52–75) in subgroups 1 and 2, respectively. Smoking habit, as well as alcohol abuse, was recorded in 9 and 6 patients. 17 nonirradiated controls. | | | MMP-20 | MMP-20 expression was pronounced and intense along the DEJ of all of the irradiated and nonirradiated examined specimens;  No differences in MMP-20 expression in the DEJ, dentin-pulp complex components, and carious dentin of post-HNRT patients |  |  |
|  |  |  |  |  |  |  |  | |  |  |  |  |  |  |  |  |  |
| Lee et al, 2013 | South Korea | 7 Carious dentin | Sound teeth | Extracted human teeth (protein extraction) |  | | Western Blot | | MMP-13 antibody was recognized in both its latent and active forms, in the crown in normal e decayed teeth;  The root in normal teeth, C1 and C2 grade caries: white bands are observed at ~50 kDa. A root with C3 grade caries; strong expression is observed at ~50 kDa. | | NA | | | MMP-13 | MMP-13 was not expressed in the coronal dentin, expressed weakly in the sound root dentin, and markedly expressed in roots with a wide range of pulp-invading caries |  |  |
| Tjaderhane et al, 1998 | Canada; Finland; England | 37 | NA | Samples of active human coronal dentinal caries lesions were collected from extracted teeth. |  | | Enzymography and Western blot | | Both MMP-2 and 9 were detected in their non-active and active forms and MMP-9 appeared to be the predominant gelatinolytic enzyme in dentin caries lesions. | | NA | | | MMP-2, MMP-8 and MMP-9 | MMP-2,8 and MMP-9 were identified in the soft dentin lesions by Western immunoblots, and the gelatinase activity was confirmed by enzymography |  |  |
| **Immunohistochemistry** | | | | | | | | | | | | | | | | | |
| **Author, year** | **Country** | **N (caries group)** | **N (control group)** | **Specimen collection** | |  | | **Clinical characteristics of the sample** | | **Type of collagenase** | | **Main Conclusions** | | | | |  |
| Boushell et al, 2011 | USA | 10 erupted 3rd molars and premolars with caries | 6 erupted 3rd molars and premolars without caries | Extracted | |  | | NA | | MMP-2 and BSP | | MMP-2 and BSP detected throughout the caries-free dentin with increased staining of the odontoblastic processes in the inner;  Sound vs. Carious = no differences;  The level of MMP-2 e BSP detection did not change with the level of caries severity. | | | | |  |
| Chibinski et al, 2014 | Brazil | 25 carious dentin of primary teeth at baseline | 25 carious dentin of primary teeth after cavity sealing (60-day sample) | Dentin excavator | |  | | N=33 patients of both genders, with age ranging from 3 to 10 years (average 6.0 ± 2.1) | | MMP-2, MMP-8 and MMP-9 | | Presence of the MMPs at baseline and after cavity sealing;  The expression of the MMPs increased after sealing, but statistical differences were observed only for MMP-8. | | | | |  |
| Charadram et al, 2012 | Australia | 30 permanent molar teeth carious | 15 healthy | Extracted teeth | |  | | Were obtained from male and female patients aged 20 to 35 years | | MMP-2 | | MMP-2 was detected within dentinal tubules containing odontoblastic processes. More intense reactivity for MMP-2 was detected in the Reactionary dentin compared to the comparable layer of dentin from healthy teeth. | | | | |  |
| Gomes-Silva et al, 2017 | Brazil | 19 Irradiated carious dentin (15 male and 4 female)  Mean age: 58-60 years | 17 Non Irradiated carious dentin | Extracted teeth (demineralized and non-demineralized) | |  | | Erupted teeth (n=36) from HNC patients. 19 post-HNRT.  Smoking habit, as well as alcohol abuse, was recorded in 9 and 6 patients. | | MMP-2 and 9 | | The MMP-2 and MMP-9 expression levels were pronounced and intense along the DEJ in all specimens;  MMP-2 and MMP-9 highly positive in carious dentin;  Tertiary dentine, pre-dentine and pulp were variably positive, and MMP-9 was predominantly positive in the non-irradiated specimens. | | | | |  |
| Kuhn et al, 2016 | Brazil | 23 Carious dentin | 23 mesial portions from carious lesion | Dentin excavator | |  | | Students of both genders with ages ranging from 7 to 15 years (11.0 +-2.7 years). | | MMP-2, 8 e 9 | | MMP-8 was reduced after 60 days of sealing, and no difference was observed for MMP-2 and MMP-9;  The MMPs’ distribution was generalized in the intertubular dentin and absent or located in the intratubular dentin, regardless of the period. | | | | |  |
| Loreto et al., 2014 | Brazil | 10 Carious dentin | 2 Sound 3rd molars | Extracted teeth (specimens were demineralized) | |  | | NA | | MMP-13 | | Sound dentin exhibited very weak immunoreactivity that was detected only at the peritubular level;  On the contrary dilated dentinal tubuli close to the caries process showed very strong immunoreactivity;  MMP-13 immunostaining diminished with increasing distance from the caries process. | | | | |  |
| Nascimento et al, 2011 | Brazil | 8 Carious dentin | 4 Sound third molar | Extracted teeth | |  | | Adults (20 to 30 years) | | Cathepsin B | | Intense and consistent cathepsin B immunostaining was observed in odontoblasts and dentinal tubules of caries lesions with less intensity in healthy teeth, and no staining in negative controls. | | | | |  |
|  |  | 42 chronic or active caries lesions | NA | Sterile spoon excavators | |  | |  | | Cysteine cathepsin proteinases /Does not specify the MMP group | | A statistically significant increase in cysteine proteinase activity was observed with the increasing depth of the dentinal caries lesions. | | | | |  |
|  |  |  |  |  |  |  |  |  |  |  |  | The negative correlations between the age and enzyme activities in active caries lesions were strong for both the cysteine cathepsins and MMPs. | | | | |  |
|  |  |  |  |  |  |  |  |  |  |  |  | A strong positive correlation was observed with cysteine cathepsin and MMP activities | | | | |  |
| Shimada, Y 2009 | Japan | 5 third molar with Carious dentin | NA | Extracted | |  | | NA | | MMP-2, MMP-8, MMP-9 e MMP-20 | | MMP-2 was distributed in both carious and sound dentin; the level of MMP-2 showed no significant difference among the outer carious, inner carious, and sound dentine;  Other MMPs showed a significant difference of distribution between different dentine regions: MMP-8 and MMP-9 increased at the outer caries may be from saliva and might cause the breakdown of dentine matrix in the outer caries lesion | | | | |  |
| Toledano, M. 2010 | Spain | 10 Carious dentin | Sound dentin | Extracted | |  | | Teeth were collected from patients aged from 18 to 20 years | | MMP-2 | | MMP-2 was present in both coronal and radicular dentin of all 10 specimens, but the immunoreactivity to MMP-2 varied markedly within the different dentin regions;  More intense immunoreactivity was coincident with areas surrounding wider dentinal tubules that resulted from the mineral dissolution during the carious process;  Adjacent to the “caries-infected” dentin, a zone of “caries affected” dentin could be identified that exhibited a low intensity of MMP-2 expression than the caries-infected dentine, but with much higher immunoreactivity than sound dentine | | | | |  |
| Vidal et al, 2014 | Brazil | 5 Carious dentin | 5 Sound dentin | Extracted | |  | | 3rd molars from individuals that were aged from 25 to 38 years. | | Catepsina B e K e MMP-2 e 9 | | CTs and MMPs, were more intensely localized in regions that correspond to the pulp chamber, predentin, and/or inner dentin;  Immunodetection of proteases was markedly higher in caries than in sound dentin; | | | | |  |
|  |  |  |  |  |  |  |  |  |  |  |  | Abundance of CT-B and CT-K was six- and seven-fold higher, respectively, in caries than in sound dentin; abundance of MMP-2 and MMP-9 was, respectively, 5- and 15-fold higher in caries affected dentin than sound dentin | | | | |  |
| ELISA | | | | | | | | | | | | | | | | | |
| **Author, year** | **Country** | **N (caries group)** | **N (control group)** | **Specimen collection** | |  | | **Clinical characteristics of the sample** | | | |  | **Main Conclusions** | | | |  |
| Ballal et al, 2017 | Switzerland | 33 teeth | NA | Paper point | |  | | Clinically healthy patients, 13 females and 17 males, aged 18–47 years (mean = 25 years, median = 27 years). | | | |  | Significantly more MMP-9 in deep carious lesions; No difference for MMP-2 | | | |  |

**Supplementary Appendix 5.** Data collection from studies evaluating bacterial collagenases

| Author, year | Country | N (caries group) | N (control group) | Specimen collection |  | Methods | Collagenase assay OD or gene expression | Clinical characteristics of the sample | Type of collagenase | Main Conclusions |
| --- | --- | --- | --- | --- | --- | --- | --- | --- | --- | --- |
| Arroyo and Simón Soro, 2015 | Spain | 3 | NA | Drill |  | Collagenase activity assays for selected isolates (ELISA) | Fluorescence measurements provided by Tecan:  *Porphyromonas gingivalis (11700)*  *Lactobacillus delbrueckii (3543.6)*  *Lactobacillus gasseri (3861.5)*  *Lactobacillus rhamnosus (4339.8)*  *Lactobacillus salivarius (3634.5)*  *Lactobacullus zeae (3720.9)*  *Prevotella denticola (3575.0)*  *Intermediate prevotel (3542.5)*  *Pseudoramibacter alactolyticus (2752.4)*  *Streptococcus mutans (2968.0)*  *Streptococcus salivarius (3033.1)* | NA | NA | No collagenolytic activity, due to the low concentration of collagen in the culture media used. |
| Hashimoto et al, 2011 | Japan | 6 subjects/ samples | NA | Curette |  | SDS-PAGE | The SDS-PAGE analysis of collagen degradation by representative protein-degrading bacteria (*P. acnes, Actinobaculum sp. oral clone EL030 and T. denticola*) showed that the collagen bands (ca. 200 and 130 kDa) were faded out during a 6-hour incubation, and several small peptide bands appeared (ca. 70, 25 and 10 KDa in *P. acnes*, ca. 17 KDa *in Actinobaculum sp*. oral clone EL030, and smear bands in T*.denticola*). | Two females and four males, age; 48-73 years; mean age 65.5 years. | NA | The proportion of protein-degrading in root caries was 7%.  *Prevotella, Actinobaculum* and *Propionibacterium* were predominant in protein-degrading isolates.  SDS-PAGE = protein-degrading bacteria isolated from plaque on root caries lesions were capable of degrading collagen;  Protein-coagulating bacteria did not degrade collagen, but produced enough organic acids to denature proteins, i.e., alter protein conformation. |

| Damé-Teixeira et al, 2018 | Brazil and United Kingdom | 30 Root caries | 10 Biofilms from sound root surfaces | Curette |  | Metatranscriptomics | The genes with the highest expression in RC were: *S. mutans* [SMU_761 and SMU_759] from and *V. parvula* [RS05935]*, P . alactolítico* [HMPREF0721_RS02020], *S. inopinata* JCM 12537 [SCIP_RS02440], *P. alactolyticus* [HMPREF0721_RS04640], and *O. uli* DSM7084 [OLSU_RS02990] | NA | Peptidase U32 Collagenase-like protease, PrtC family | 201 genes coding for bacterial collagenolytic proteases were identified in 113 bacterial species; 24 from *Prevotella* spp. and 20 from *Streptococcus* spp.  42 bacterial collagenolytic proteases with significant differential expression: 24 were overexpressed in SRS and 18 in RC |
| --- | --- | --- | --- | --- | --- | --- | --- | --- | --- | --- |
|  |  |  |  |  |  |  | In SRC: *L. buccalis [*LEBU_RS10190 and LEBU_RS05040]  Overexpressed proteases in RC: *P. alactolyticus* [HMPREF0721_RS02020], *S. inopinata* JCM 12537 [SCIP_RS02440], *P. alactolyticus* [HMPREF0721_RS04640], and *O. uli* DSM7084 [OLSU_RS02990] |  |  |  |
|  |  |  |  |  |  |  |  |  |  |  |
| Simon-Soro et al. 2013 | Spain | 3  dentin caries | 3  supragengival dental plaque from enamel lesions | Excavator |  | Metagenomics | NA | NA | NA | Genes coding for collagenases and other proteases enabling dentin degradation are significantly overrepresented in dentin cavities. |

NA=Not available
