# Supplementary material for: Presence of host and bacterial-derived collagenolytic proteases in carious dentin: a systematic review of ex vivo studies
Source: Front Cell Infect Microbiol. 2023 Oct 31;13:1278754. doi: 10.3389/fcimb.2023.1278754 (PMC10644316; doi:10.3389/fcimb.2023.1278754)
Supplement: Supplementary file 2 [file Table_2.docx]

***Presence of host and bacterial-derived collagenolytic proteases in carious dentin: a systematic review of ex vivo studies***

**Supplementary Appendix 6.** Certainty of body of evidence assessed through the GRADE approach (GRADEpro tool) for reviews without metanalysis (MMP-2, MMP-8, MMP-9, MMP-13, MMP-20, and CT-B)

**6.1. Question:** Caries in dentin and/or root surface compared to sound dentin for presence of **MMP-2**

| **Certainty assessment** | | | | | | | **Impact** | **Certainty** | **Importance** |
| --- | --- | --- | --- | --- | --- | --- | --- | --- | --- |
| **№ of studies** | **Study design** | **Risk of bias** | **Inconsistency** | **Indirectness** | **Imprecision** | **Other considerations** |  |  |  |
| **Sound vs. Carious** | | | | | | | | | |
| 7^1,2,3,4,5, 6,7^ | observational studies | serious^a^ | not serious | not serious | serious^b^ | none | MMP-2 may be increased in carious dentin when compared to sound dentin, but the evidence is very uncertain. | ⨁◯◯◯ Very low |  |
| **Coronal vs. root dentin** | | | | | | | | | |
| 1^2^ | observational studies | serious^c^ | not serious | not serious^d^ | serious^e^ | none | There is no difference of MMP-2 expression/activity in root versus coronal carious dentin, but evidence is very uncertain. | ⨁◯◯◯ Very low |  |
| **Lesion depth and location** | | | | | | | | | |
| 5^1 3,4,6,8^ | observational studies | not serious^f^ | serious | not serious | serious^b^ | none | The evidence is very uncertain about the MMP-2 location in dentin, as the studies show conflicting results and no effect direction was found. | ⨁◯◯◯ Very low |  |
| **Before and after cavity sealing** | | | | | | | | | |
| 2^9,10^ | observational studies | not serious | not serious | not serious | serious^e^ | none | There is no difference of MMP-2 expression/activity before and after cavity sealing, but the evidence is very uncertain. | ⨁◯◯◯ Very low |  |

#### Explanations

a. Low methodological quality of 3 out of 6 studies considering the Joanna Briggs Institute instrument.

b. Did not reach the optimal information size (OIS) (N=244 samples for sound vs. carious and N=440 for lesion depth and location, calculated using the OpenEpi.com tool, based on averages from Shimada et al. 2009 – labelling index differences of 0.92 - and median values from Ballal et al. 2017 – labelling index differences of 1.5 –; confidence intervals of 95%, and a power of 80%).

c. Low methodological quality of the study considering the Joanna Briggs Institute instrument.

d. Lack of evidence about generalization and external validity.

e. Did not reach the OIS of the GRADE (less than 800 samples/N; no information in the literature to calculate a specific OIS).

f. Low methodological quality of 1 out of 2 studies considering the Joanna Briggs Institute instrument.

#### References

1.CM, Vidal, L, Tjäderhane, PM, Scaffa, IL, Tersariol, D, Pashley, HB, Nader, FD, Nascimento, MR, Carrilho. Abundance of MMPs and cysteine cathepsins in caries-affected dentin.. Journal of dental research; 2014.

2.M, Toledano, R, Nieto-Aguilar, R, Osorio, A, Campos, E, Osorio, FR, Tay, M, Alaminos. Differential expression of matrix metalloproteinase-2 in human coronal and . Journal of dentistry; 2010.

3.LW, Boushell, H, Nagaoka, H, Nagaoka, M, Yamauchi. Increased matrix metalloproteinase-2 and bone sialoprotein response to human . Caries research; 2011.

4. Y, Shimada, S, Ichinose, A, Sadr, M, Burrow, J, Tagami. Localization of matrix metalloproteinases (MMPs-2, 8, 9 and 20) in normal and carious dentine. Australian dental journal; 2009.

5. L, Tjäderhane, H, Larjava, T, Sorsa, VJ, Uitto, M, Larmas, T, Salo.The Activation and Function of Host Matrix Metalloproteinases in Dentin Matrix Breakdown in Caries Lesions. Journal of Dental Research ; 1998.

6.W, Gomes-Silva, AC, Prado,Ribeiro, G, de,Castro,Junior, JV, Salvajoli, N, Rangel,Palmier, MA, Lopes, MM, Rocha, MF, de,Goes, TB, Brandão, AR, Santos-Silva. Head and neck radiotherapy does not increase gelatinase (metalloproteinase-2 and . Oral surgery, oral medicine, oral pathology and oral radiology; 2017.

7. [N, Charadram](https://pubmed.ncbi.nlm.nih.gov/?term=Charadram+N&cauthor_id=22079283), [RM, Farahani](https://pubmed.ncbi.nlm.nih.gov/?term=Farahani+RM&cauthor_id=22079283), [D,Harty](https://pubmed.ncbi.nlm.nih.gov/?term=Harty+D&cauthor_id=22079283), [C, Rathsam](https://pubmed.ncbi.nlm.nih.gov/?term=Rathsam+C&cauthor_id=22079283), [MV, Swain](https://pubmed.ncbi.nlm.nih.gov/?term=Swain+MV&cauthor_id=22079283), [N, Hunter](https://pubmed.ncbi.nlm.nih.gov/?term=Hunter+N&cauthor_id=22079283). Regulation of reactionary dentin formation by odontoblasts in response to polymicrobial invasion of dentin matrix. Bone; 2012.

8.V, Ballal, S, Rao, A, Bagheri, V, Bhat, T, Attin, M, Zehnder. MMP-9 in Dentinal Fluid Correlates with Caries Lesion Depth.. Caries research; 2017.

9.E, Kuhn, A, Reis, EB, Campagnoli, AC, Chibinski, MR, Carrilho, DS, Wambier. Effect of sealing infected dentin with glass ionomer cement on the abundance and . International journal of paediatric dentistry; 2016.

10.AC, Chibinski, JR, Gomes, K, Camargo, A, Reis, DS, Wambier. Bone sialoprotein, matrix metalloproteinases and type I collagen expression after . Caries research; 2014.

**6.2. Question:** Caries in dentin and/or root surface compared to sound dentin for **MMP-8**

| **Certainty assessment** | | | | | | | **Impact** | **Certainty** | **Importance** |
| --- | --- | --- | --- | --- | --- | --- | --- | --- | --- |
| **№ of studies** | **Study design** | **Risk of bias** | **Inconsistency** | **Indirectness** | **Imprecision** | **Other considerations** |  |  |  |
| **Sound vs. Carious dentin** | | | | | | | | | |
| 2^1,2^ | observational studies | not serious | not serious | not serious | serious^a^ | none | The evidence is very uncertain about the MMP-8 expression in sound versus carious dentin, as the included studies show conflicting results and no effect direction was found. | ⨁◯◯◯ Very low |  |
| **Before vs. after cavity sealing** | | | | | | | | | |
| 2^3,4^ | observational studies | not serious | not serious | not serious | serious^b^ | none | MMP-8 may be increased in the dentin before when compared to dentin after cavity sealing, but the evidence is very uncertain. | ⨁◯◯◯ Very low |  |

#### Explanations

a. Did not reach the OIS (N=26 samples, calculated using the OpenEpi.com tool, based on averages from Shimada et al. 2009 – labelling index differences of 3.42; confidence intervals of 95%, and a power of 80%).

b. Did not reach the OIS of GRADE (less than 800 samples/N; no information in the literature to calculate a specific OIS).

#### References

1. Y, Shimada, S, Ichinose, A, Sadr, M, Burrow, J, Tagami. Localization of matrix metalloproteinases (MMPs-2, 8, 9 and 20) in normal and carious dentine. Australian dental journal; 2009.

2. L, Tjäderhane , H, Larjava, T, Sorsa,, VJ, Uitto, M, Larmas, T, Salo. The Activation and Function of Host Matrix Metalloproteinases in Dentin Matrix Breakdown in Caries Lesions. Journal of Dental Research; 1998.

3.AC, Chibinski, JR, Gomes, K, Camargo, A, Reis, DS, Wambier. Bone sialoprotein, matrix metalloproteinases and type I collagen expression after . Caries research; 2014.

4.E, Kuhn, A, Reis, EB, Campagnoli, AC, Chibinski, MR, Carrilho, DS, Wambier. Effect of sealing infected dentin with glass ionomer cement on the abundance and . International journal of paediatric dentistry; 2016.

**6.3. Question:** Caries in dentin and/or root surface compared to sound dentin for **MMP-9**

| **Certainty assessment** | | | | | | | **Impact** | **Certainty** | **Importance** |
| --- | --- | --- | --- | --- | --- | --- | --- | --- | --- |
| **№ of studies** | **Study design** | **Risk of bias** | **Inconsistency** | **Indirectness** | **Imprecision** | **Other considerations** |  |  |  |
| **Sound vs. Carious dentin** | | | | | | | | | |
| 4^1,2,3,4^ | observational studies | serious^a^ | serious^b^ | not serious | serious^c^ | none | MMP-9 may be increased in carious dentin when compared to sound, but the evidence is very uncertain. | ⨁◯◯◯ Very low |  |
| **Lesion depth and location** | | | | | | | | | |
| 2^3,5^ | observational studies | serious | serious | not serious | serious^d^ | none | The evidence is very uncertain about the MMP-9 expression in different locations and lesion depth, as the studies show conflicting results and no effect direction was found. | ⨁◯◯◯ Very low |  |
| **Before vs. after cavity sealing** | | | | | | | | | |
| 2^6,7^ | observational studies | not serious | not serious | not serious | serious^e^ | none | There is no difference of MMP-9 expression/activity before and after cavity sealing, but the evidence is very uncertain. | ⨁◯◯◯ Very low |  |

#### Explanations

a. Low methodological quality of the study considering the Joanna Briggs Institute instrument.

b. Of the 4 included studies, 1 found significantly more MMP-9 in carious dentin than sound, 2 observed its presence also in carious dentin, but without statistical calculation and without comparison with sound tissue, and 1 found more in sound tissue (p<0.05).

c. Did no reach the OIS (N=62 samples, calculated using the OpenEpi.com tool, based on averages from Shimada et al. 2009 – labelling index average difference of 2; confidence intervals of 95%, and a power of 80%).

d. Reached the OIS (N=14 samples, calculated using the OpenEpi.com tool, based on averages from Shimada et al. 2009 – labelling index average difference of -3.5; confidence intervals of 95%, and a power of 80%).

e. Did not reach the OIS of GRADE (less than 800 samples/N; no information in the literature to calculate a specific OIS).

#### References

1.W, Gomes-Silva, AC, Prado,Ribeiro, G, de,Castro,Junior, JV, Salvajoli, N, Rangel,Palmier, MA, Lopes, MM, Rocha, MF, de,Goes, TB, Brandão, AR, Santos-Silva. Head and neck radiotherapy does not increase gelatinase (metalloproteinase-2 and . Oral surgery, oral medicine, oral pathology and oral radiology; 2017.

2.CM, Vidal, L, Tjäderhane, PM, Scaffa, IL, Tersariol, D, Pashley, HB, Nader, FD, Nascimento, MR, Carrilho. Abundance of MMPs and cysteine cathepsins in caries-affected dentin.. Journal of dental research; 2014.

3. Y, Shimada, S, Ichinose, A, Sadr, M, Burrow, J, Tagami. Localization of matrix metalloproteinases (MMPs-2, 8, 9 and 20) in normal and carious dentine. Australian dental journal; 2009.

4. Tjäderhane L, Larjava, H, Sorsa, T, Uitto, VJ, Larmas, M, Salo. The Activation and Function of Host Matrix Metalloproteinases in Dentin Matrix Breakdown in Caries Lesions. Journal of Dental Research ; 1998.

5.V, Ballal, S, Rao, A, Bagheri, V, Bhat, T, Attin, M, Zehnder. MMP-9 in Dentinal Fluid Correlates with Caries Lesion Depth.. Caries research; 2017.

6.AC, Chibinski, JR, Gomes, K, Camargo, A, Reis, DS, Wambier. Bone sialoprotein, matrix metalloproteinases and type I collagen expression after . Caries research; 2014.

7.E, Kuhn, A, Reis, EB, Campagnoli, AC, Chibinski, MR, Carrilho, DS, Wambier. Effect of sealing infected dentin with glass ionomer cement on the abundance and . International journal of paediatric dentistry; 2016.

**6.4. Question:** Caries in dentin and/or root surface compared to sound dentin for **MMP-13**

| **Certainty assessment** | | | | | | | **Impact** | **Certainty** | **Importance** |
| --- | --- | --- | --- | --- | --- | --- | --- | --- | --- |
| **№ of studies** | **Study design** | **Risk of bias** | **Inconsistency** | **Indirectness** | **Imprecision** | **Other considerations** |  |  |  |
| **Sound vs. Carious dentin** | | | | | | | | | |
| 1^1^ | observational studies | not serious | not serious | not serious | serious^a^ | none | MMP-13 may be increased in carious dentin when compared to sound, but the evidence is very uncertain. | ⨁◯◯◯ Very low |  |
| **Coronal vs. Root** | | | | | | | | | |
| 1^2^ | observational studies | not serious | not serious | not serious | serious^a^ | none | MMP-13 may be increased in root caries when compared to coronal, but the evidence is very uncertain. | ⨁◯◯◯ Very low |  |

#### Explanations

a. Did not reach the optimal information size of GRADE (less than 800 samples/N; no information in the literature to calculate a specific OIS).

#### References

1. [C,Loreto](https://pubmed.ncbi.nlm.nih.gov/?term=Loreto%20C%5BAuthor%5D), [C, Galanti](https://pubmed.ncbi.nlm.nih.gov/?term=Galanti%20C%5BAuthor%5D), [G, Musumeci](https://pubmed.ncbi.nlm.nih.gov/?term=Musumeci%20G%5BAuthor%5D), [MC, Rusu](https://pubmed.ncbi.nlm.nih.gov/?term=Rusu%20M%5BAuthor%5D), [R, Leonardi](https://pubmed.ncbi.nlm.nih.gov/?term=Leonardi%20R%5BAuthor%5D). Immunohistochemical Analysis of Matrix Metalloproteinase-13 in Human Caries Dentin. European journal of histochemistry: EJH; 2014.

2.T, Lee, E, Jin, B, Choi. MMP-13 expression in coronal and radicular dentin according to caries progression - A pilot study. Tissue Engineering and Regenerative Medicine; 2013.

**6.5. Question:** Caries in dentin and/or root surface compared to sound dentin for **MMP-20**

| **Certainty assessment** | | | | | | | | | | | | | | **Impact** | **Certainty** | | | **Importance** | |  |
| --- | --- | --- | --- | --- | --- | --- | --- | --- | --- | --- | --- | --- | --- | --- | --- | --- | --- | --- | --- | --- |
| **№ of studies** | | **Study design** | | **Risk of bias** | | **Inconsistency** | | **Indirectness** | | **Imprecision** | | **Other considerations** | |  |  |  |  |  |  |  |
| **Lesion depth and location** | | | | | | | | | | | | | | | | | | | |  |
| 1^2^ | | observational studies | | not serious | | not serious | | not serious | | not serious^a^ | | none | | MMP-20 probably reduces in carious outer dentin when compared to carious inner dentin. | | ⨁⨁⨁◯  Modarate |  | | |  |
| **Irradiated vs. non irradiated** | | | | | | | | | | | | | | | | | | | | |
| 1^1^ | | observational studies | | not serious | | not serious | | not serious | | serious^b^ | | none | | There is no difference about the MMP-20 expression in irradiated vs. non irradiated carious dentin, but the evidence is very uncertain. | | ⨁◯◯◯ Very low | | | |  |

#### Explanations

1. Reached the OIS (N=4, sample size calculated using the OpenEpi.com tool, based on from Shimada et al. 2009 – difference in labeling index differences of -5.72, confidence interval of 95%, and a power of 80%).
2. Did not reach the optimal information size of GRADE (less than 800 samples/N; no information in the literature to calculate a specific OIS).

#### References

1.W, da Silva, AC, Ribeiro, T, Brandão, K, Morais-Faria, G, Castro Junior, M, Mak, M, Lopes, M, Rocha, T, Salo, L, Tjäderhane, M, Goes, A, Santos-Silva. Postradiation Matrix Metalloproteinase-20 Expression and Its Impact on Dental Micromorphology and Radiation-Related Caries. Caries research; 2017.

2. Y, Shimada, S, Ichinose, A, Sadr, M, Burrow, J, Tagami. Localization of matrix metalloproteinases (MMPs-2, 8, 9 and 20) in normal and carious dentine. Australian dental journal; 2009.

**6.6. Question:** Caries in dentin and/or root surface compared to sound dentin for CT-B

| **Certainty assessment** | | | | | | | **Impact** | **Certainty** | **Importance** |
| --- | --- | --- | --- | --- | --- | --- | --- | --- | --- |
| **№ of studies** | **Study design** | **Risk of bias** | **Inconsistency** | **Indirectness** | **Imprecision** | **Other considerations** |  |  |  |
| **Sound vs. Carious dentin** | | | | | | | | | |
| 2^1,2^ | observational studies | serious^a^ | not serious | not serious | not serious^b^ | none | CT-B expression may be increased in carious dentin when compared to sound dentin, but the evidence is very uncertain. | ⨁◯◯◯ Very low |  |

#### Explanations

a. Low and moderate methodological quality considering the Joanna Briggs Institute instrument.

b. Reached the optimal information size (sample size calculated using the OpenEpi.com tool, based on approximate averages of labeling index from Nascimento et al., 2011, confidence interval of 95%, and a power of 80%).

#### References

1.CM, Vidal, L, Tjäderhane, PM, Scaffa, IL, Tersariol, D, Pashley, HB, Nader, FD, Nascimento, MR, Carrilho. Abundance of MMPs and cysteine cathepsins in caries-affected dentin.. Journal of dental research; 2014.

2. F, Nascimento, CL, Minciotti, S, Geraldeli, M, Carrilho, DH, Pashley, F, Tay, H, Nader, T, Salo, L, Tjäderhane, I, Tersariol. Cysteine Cathepsins in Human Carious Dentin. Journal of dental research; 2011.
